# Supplementary material for: Probe exciplex structure of highly efficient thermally activated delayed fluorescence organic light emitting diodes
Source: Nat Commun. 2018 Aug 6;9:3111. doi: 10.1038/s41467-018-05527-4 (PMC6079109; doi:10.1038/s41467-018-05527-4)
Supplement: Supplementary file 1 — Supplementary Information [file 41467_2018_5527_MOESM1_ESM.pdf]

Supplementary information

Probe Exciplex Structure of Highly Efficient Thermally Activated Delayed Fluorescence Organic Light Emitting Diodes

Lin et al.

Contents		Page
**Supplementary Methods**		**S4**
**Supplementary Table 1**	Crystal data and structure refinement parameters for CN-Cz2.	**S10**
**Supplementary Table 2**	EL characteristics of exciplex-based OLEDs.	**S11**
**Supplementary Table 3**	Vibration peaks and lifetimes of CN-Cz2, PO-T2T and CN-Cz2:PO-T2T in Step-Scan spectra.	**S11**
**Supplementary Figure 1**	Synthesis of CN-Cz2.	**S12**
**Supplementary Figure 2**	Thermal ellipsoidal plot of CN-Cz2 (50% probability).	**S12**
**Supplementary Figure 3**	Cyclic voltammograms of CN-Cz2.	**S13**
**Supplementary Figure 4**	Representative TOF transients of CN-Cz2 for **a.** holes (linear), **b.** holes (log10), **c**. electrons (linear) and **d**. electrons (log10) at E = 1.0 × 10^6 V/cm. **e.** Carrier mobility of CN-Cz2 plotted with respect to $E^{1/2}$.	**S14**
**Supplementary Figure 5**	HOMO energy level of CN-Cz2 determined by using photoelectron yield spectroscopy.	**S15**
**Supplementary Figure 6**	PLQY for CN-Cz2:PO-T2T with different weight ratio.	**S15**
**Supplementary Figure 7**	Device structure of the CN-Cz2:PO-T2T exciplex OLED.	**S16**
**Supplementary Figure 8**	The performance parameters of OLEDs.	**S16**
**Supplementary Figure 9**	The setup of Step-Scan FTIR experiment.	**S17**
**Supplementary Figure 10**	The steady-state IR spectra of CN-Cz2, PO-T2T and CN-Cz2:PO-T2T.	**S17**
**Supplementary Figure 11**	The transient IR character. The decay profiles of new generated exciplex band (1319, 1547, 1570 cm^{-1} and band ~2060 cm^{-1}) of CN-Cz2:PO-T2T exciplex.	**S18**
**Supplementary Figure 12**	The transient IR character. The decay (2214 cm^{-1} peak) and rise (1597, 2221 cm^{-1} peak)	**S19**

	dynamics of CN-Cz2.	
**Supplementary Figure 13**	The transient IR character. The decay (1512, 1562 cm^{-1} peak) and rise (1527 cm^{-1} peak) dynamics of PO-T2T.	**S20**
**Supplementary Figure 14**	The structure of molecular packing and polaron formation.	**S21**
**Supplementary Figure 15**	The transient IR character. Absorbance-frequency-time 3D plot for the CN-Cz2:PO-T2T with different weight ratio **a.**(2:1), **b.** (1:1) and **c.** (1:2).	**S22**
**Supplementary Figure 16**	The transient IR character of exciplex composed of NPNPB and PO-T2T.	**S23**
**Supplementary Figure 17**	Photophysical property of PXZ-TRZ.	**S24**
**Supplementary Figure 18**	The transient IR character of PXZ-TRZ. a. An absorbance-frequency-time 3D plot for the transient IR of PXZ-TRZ film.	**S25**
**Supplementary Figure 19**	Photophysical property of DMAC-TRZ.	**S26**
**Supplementary Figure 20**	The transient IR character of DMAC-TRZ.	**S27**
**Supplementary Figure 21**	Photophysical property and the transient IR character of PXZPBM.	**S28**
**Supplementary Figure 22**	The transient IR character. The decay 1508 cm^{-1} and rise (1525, 1597, 2221 cm^{-1}) dynamics of CN-Cz2:PO-T2T exciplex.	**S29**
**Supplementary Figure 23**	Transient PL decay properties of CN-Cz2:PO-T2T film. Prompt (delay = 0, gate width = 10 ns) and delayed (delay = 10 μs, gate width = 2 μs) components of PL spectra for the CN-Cz2:PO-T2T film at 300 K. A slight red shift was resolved between the prompt and delayed components.	**S30**
**Supplementary Figure 24**	Transient EL decay properties of CN-Cz2:PO-T2T film.	**S31**
**Supplementary Figure 25**	The GIXD character. The fitted 1D GIXD of CN-Cz2 film.	**S32**
**Supplementary Figure 26**	The GIXD character. Fitted 1D GIXD of co-deposited film.	**S32**
**Supplementary Figure 27**	The arrangement of CN-Cz2 and PO-T2T to form the exciplex.	**S33**
**Supplementary Figure 28**	Theoretical calculations based on the type I arrangement of CN-Cz2 and PO-T2T. The optimized structure of S_1 for the type I of CN-Cz2:PO-T2T exciplex in dichloromethane.	**S34**
**Supplementary Figure 29**	Theoretical calculations based on the type I arrangement of CN-Cz2 and PO-T2T. The	**S35**

	optimized structure of T_1 for the type I of CN-Cz2:PO-T2T exciplex in dichloromethane.	
**Supplementary Figure 30**	Theoretical calculations based on the type II arrangement of CN-Cz2 and PO-T2T. The optimized structure of S_1 for the type II of CN-Cz2:PO-T2T exciplex in dichloromethane.	**S36**
**Supplementary Figure 31**	Theoretical calculations based on the type II arrangement of CN-Cz2 and PO-T2T. The optimized structure of T_1 for the type II of CN-Cz2:PO-T2T exciplex in dichloromethane.	**S37**
**Supplementary Figure 32**	The calculated IR spectra of CN-Cz2:PO-T2T exciplex with type I configurations in dichloromethane.	**S38**
**Supplementary Figure 33**	Comparing the IR peaks between experiments and theoretical calculations.	**S39**
**Supplementary Figure 34**	Theoretical calculation of IR vibrational modes. (a)-(g) The calculated simultaneously IR vibrational modes for the S_1 of type I in CN-Cz2:PO-T2T exciplex within 1200 to 1600 cm^{-1} in dichloromethane.	**S40**
**Supplementary Figure 35**	Theoretical calculation of IR vibrational modes. (a)-(g) The calculated simultaneously IR vibrational modes for T_1 of type I in CN-Cz2:PO-T2T exciplex within 1200 to 1600 cm^{-1} in dichloromethane.	**S41**
**Supplementary References**		**S42**

Supplementary Methods

Synthesis

CN-Cz2 was synthesized in good yield following the synthesis shown in Supplementary Scheme 1. Formylation of at the 3-position of 9-phenyl-9H-carbazole via Vilsmeyer-Haack reaction followed by its cyanation gave the intermediate, 9-phenyl-9H-carbazole-3-carbonitrile. This intermediate was then subjected to mono-bromination at 0 ^{o}C to give compound 6-bromo-9-phenyl-9H-carbazole-3-carbonitrile in 85% yield. Finally, Ullman coupling between the bromo-intermediate and carbazole under solvent free condition in a sealed tube yielded the product 9-phenyl-9H-3,9'-bicarbazole-6-carbonitrile (CN-Cz2) in moderate yield.

Synthesis of 9-phenyl-9H-3,9'-bicarbazole-6-carbonitrile (CN-Cz2)

420 mg (1.2 mmol) of 6-bromo-9-phenyl-9H-carbazole-3-carbonitrile, 168 mg (1 mmol) carbazole, 28 mg (0.12 mmol CuI, 10 mol%), 555 mg (4 mmol) K_2CO_3, 66 mg $18C_6$, DMPU (cat. amount) were mixed in a sealed tube, evacuated and filled with argon gas. The mixture was heated at 220 ^{o}C for 48 h. After the reaction was complete, it was cooled to room temperature, neutralized with HCl and extracted with DCM. The crude mixture was subjected to silica gel column chromatography with 40:60% DCM-hexane as the eluent to get the product as a white solid. ^{1}H NMR (400 MHz, CD_2Cl_2) δ 8.44 (d, *J* = 1.2 Hz, 1H), 8.33 (d, *J* = 2.0 Hz, 1H), 8.19 (d, *J* = 8.0 Hz, 2H), 7.73 – 7.69 (m, 3H), 7.66 – 7.59 (m, 5H), 7.48(d, *J* = 8.0 Hz, 1H), 7.44-7.38 (m, 4H), 7.34-7.30 (m, 2H); ^{13}C NMR (100 MHz, $CDCl_3$) δ 143.3, 141.7, 140.7, 136.3, 131.3, 130.4, 129.8, 128.8, 127.2, 126.9, 125.9, 125.6, 123.3, 123.1, 120.3, 119.9, 119.8, 119.7, 111.6, 110.9, 109.6, 103.4; HRMS (m/z, ESI) calcd. for $C_{31}H_{19}N_3$ 433.1579. Found: 434.1652 $(M+H)^+$; Anal. calcd. for $C_{31}H_{19}N_3$: C, 85.89; H, 4.42; N, 9.69. Found C, 85.32; H, 4.38; N, 9.65.

Characterization of CN-Cz2

CN-Cz2 was synthesized in four steps, starting from 9-phenyl-9H-carbazole, which has been well characterized, including with a single X-ray structure (Supplementary Figure 1 and Supplementary Table 1). The cyclic voltammetry (CV) of CN-Cz2 showed quasi-

reversible oxidation and irreversible reduction behaviors, indicating a moderate bipolar character (Supplementary Figure 2). The density function theory (DFT) calculation reveals that the HOMO of CN-Cz2 resides on the peripheral carbazole, with limited contribution from the central carbazole, while the LUMO is located on the π*orbitals of the central CN-substituted carbazole core (Fig. 1b). Differential scanning calorimetry (DSC) and thermogravimetric analysis (TGA) analyses of CN-Cz2 were conducted to give a glass transition temperature (T_g) of 119 °C and a decomposition temperature (T_d, 5% weight loss) of 315 °C, indicating the high morphological/thermal stability and good tendency for forming amorphous films via thermal evaporation. The HOMO energy level of -5.70 eV was determined by photoemission spectrometer (PES) with a CN-Cz2 film (Supplementary Figure 3). Exploiting the time-of-flight (TOF) technique, we confirmed the bipolar charge transporting characters of CN-Cz2 by observing both electron and hole mobilities, as shown in Supplementary Figure 4.

Pump-probe time-resolved IR measurement

Step-Scan FTIR spectroscopy: Step-Scan FTIR spectroscopy was performed using a Vertex 80 spectrometer (Bruker) with an HgCdTe (MCT) detectors. Sample containing a mixture of CN-Cz2 and PO-T2T in 1:1 molar ratio was vacuum deposited on the CaF_2 substrates (one inch in diameter) to form a smooth and homogeneous film with a thickness of about 1 µm. CaF_2 was cleaned by methanol prior to the usage. In this study, a fourth harmonic of a Nd:YAG laser (Continuum, Surelite) at 266 nm was used as the pump pulse, which has a duration of 15 ns and a repetition rate of 10 Hz. This transient IR experiment was non-trivial, as both background reduction and optimization of pumping power played key roles; the former required various combinations of interference filters, and the latter was necessary to avoid thermal noise and decomposition. A typical excitation energy was adjusted from 0 to 20 mJ/cm^2. An optimized laser pulse is to have decent signal-to-noise to avoid damaging the sample. The pump laser was synchronized with the spectrometer and was set at an angle of ~45°

with respect to the IR probe beam to maximize the pump-probe overlap. During the measurement, the entire FTIR compartment was purged with nitrogen at room temperature. An interference filter (< 3300 cm^{-1}) was used in front of the MCT detector, which efficiently blocked the stray light from the excitation laser and minimized the interferogram points.

Crystal structure

Single crystals suitable for X-ray diffraction structure analysis for CN-Cz2 were obtained from the slow diffusion of n-pentane to a $CHCl_3$ solution containing the compound. It crystallizes in the triclinic space group P-1 and the thermal ellipsoidal plot of the same is presented in Supplementary Figure 1. The relevant crystal data and structure refinement parameters are given in Supplementary Table 1.The dihedral angle of the pendant carbazole unit with respect to the central phenyl-carbazole core is ~75° and is thus, out-of-plane from the central core.

Electrochemical properties

The electrochemical properties of the compound CN-Cz2 were investigated by cyclic voltammetry. The compound displays two types of oxidation peaks corresponding to the unsubstituted and substituted carbazole moieties respectively, similar to previously reported literature.[1-3] The irreversibility of the first oxidation peak might be ascribed to the presence of electrochemically active sites in the unsubstituted units thereby leading to polymerization while the substituted positions (3 or 6) of carbazole exhibit nearly reversible peaks since in this case, no C-C coupling can occur.[1]

Time-of-flight (TOF) mobility measurements

In charge-transport properties of CN-Cz2, we used the terfluorene (E3) as the charge-generation layer (CGL) for the time-of-flight (TOF) transient photocurrent technique.[4] The TOF device was configured as: ITO glass/E3 (0.1 μm)/ CN-Cz2 (1.1 μm)/Ag (150 nm), which were then placed inside a cryostat and kept under vacuum. The thickness of

organic film was monitored in situ with a quartz sensor and calibrated by a thin film thickness measurement (K-MAC ST2000). A pulsed nitrogen laser (337 nm) was used as the excitation light source through the transparent electrode (ITO) inducing photogeneration of a thin sheet of excess carriers. Under an applied dc bias, the transient photocurrent was swept across the bulk of the organic film toward the collection electrode (Ag), and then recorded with a digital storage oscilloscope. Depending on the polarity of the applied bias, selected carriers (holes or electrons) are swept across the sample with a transit time of t_T. With the applied bias V and the sample thickness D, the applied electric field $E = V/D$, and the carrier mobility is then given by $\mu = D/(t_T E) = D^2/(Vt_T)$, in which the carrier transit time, t_T, can be extracted from the intersection of two asymptotes to the tail and plateau sections in the double-logarithmic plots.

OLED Device

All chemicals were purified through vacuum sublimation prior to use. The OLEDs were fabricated through vacuum deposition of the materials at 10^{-6} Torr onto the ITO-coated glass substrates having a sheet resistance of 15 Ω sq^{-1}. Prior to use the ITO surface was cleaned ultrasonically; i.e. with acetone, methanol, and deionized water in sequence and finally with UV-ozone. The deposition rate of each organic material was ca. 1–2 Å s^{-1}. The *J*–*V*–*L* characteristics of the devices were measured simultaneously in a glove-box using a Keithley 2614B source meter equipped with a calibration Si-photodiode. EL spectra were measured using a photodiode array (Ocean Optics USB2000+).

Grazing-incidence X-ray diffraction (GIXD) measurement.

GIXD was performed at beamline 01C2 of Taiwan Light Source at National Synchrotron Radiation Research Center (NSRRC), Taiwan. The studied thin films were deposited on the Si substrate. The GIXD data were collected with 12 keV X-rays of a wavelength 1.033 Å by a 2D image plate (Mar345). The incidence angle of X-ray beam was ~0.2° for GIXD.

Computational Methods

All of the calculations were performed with the Gaussian 09 programpackage[5]. The geometry optimization of ground states of CN-Cz2 and PO-T2T were simulated with density functional theory (DFT) at the B3LYP/6-31G(d) levels using cyclohexane as the solvent. The solvent effect is based on the polarizable continuum model (PCM), which is implemented in the Gaussian 09 program. We also simulated the optimized structures of singlet and triplet excited states for CN-CZ2:PO-T2T. The initial positions of CN-CZ2:PO-T2T were placed as close as possible to each other and solvent cyclohexane was also used.

The computational approach to access the optimum CN-Cz2:PO-T2T exciplex

The results of GIXD lead us to propose that the contact between CN-Cz2 and PO-T2T, at the molecular level, is in an in-plane intercalation-like configuration, in which the polar-polar interaction between donor and acceptor sites plays key role rather than the π-π stacking interaction. On this basis, we then performed the ab initio computational approach. Our sequence of strategy and methodology are as follows

(1) We aligned the CN-Cz2:PO-T2T structure on the basis of in-plane, polar-polar interaction between donor and acceptor sites.

(2) To differentiate the CN-carbazole and carbazole sites in CN-Cz2, the CN- site has been intentionally placed toward or away from PO-T2T.

(3) After obtaining the locally minimized CN-Cz2:PO-T2T structure, the vertical excitation was executed, followed by the geometry optimization.

(4) We then examined the electron density distribution of the optimized exciplex, from which only considered are those with small but non-negligible overlap between HOMO and LUMO for further analyses.

As a result, to our best approaches, there are two displacement-oriented CN-Cz2:PO-T2T structures that fulfill the criteria of (1)-(3), symbolized as type I and II in Supplementary Figure 26); however, only type I orientation depicted below (Figure 3 in the text) that meets the requirement 4. As for this optimized exciplex in the T_1 state the

HOMO and LUMO are calculated to have 0.13% and 0.13% contribution from PO-T2T and CN-Cz2, respectively, indicating the slight overlap between HOMO and LUMO. We further calculated the associated IR spectra based on this exciplex configuration. The results depicted in Supplementary Figure 31 match well the transient IR spectra obtained from the Step-Scan Fourier Transform IR technique. Note that the weak interaction between PO-T2T and CN-Cz2 in the exciplex makes partial cancellation of the IR peaks between PO-T2T/CN-Cz2 exciplex (product) and PO-T2T (CN-Cz2, reactant). Nevertheless, several new peaks associated with the exciplex can be well distinguished, which, together with their relaxation dynamics, have been elaborated in the text.

Supplementary Table 1. Crystal data and structure refinement parameters for CN-Cz2.

CCDC 1547184	CN-Cz2
Emperical formula	$C_{31} H_{19} N_3$
Formula weight	433.49
T /K	123 (2)
Crystal system	Triclinic
Wavelength/ Å	0.71073
Space group	P-1
a /Å	12.1769(4)
b /Å	13.9495(5)
c /Å	14.7648(6)
α /º	78.496(3)
β /º	83.070(3)
γ /º	64.847(3)
V /Å^3	2222.73(14)
Z	4
ρ_c /Mg.m^{-3}	1.295
μ /mm^{-1}	0.077
F (000)	904
Reflections collected	24804
Unique reflections (R_{int})	9858, 0.0400
R_1, wR_2 [I>2σ(I)]	0.0500, 0.1295
R_1, wR_2 [all data]	0.0819, 0.1591
GooF on F^2	0.940
Largest diff. peak and hole/ e.Å^{-3}	0.209, -0.238

Supplementary Table 2. EL characteristics of exciplex-based OLEDs.

CN-Cz2:PO-T2T	V_{on}[V]	L_{max} [cd/m^2]	I_{max} [mA/cm^2]	EQE,CE_{max} [%,cd/A]	PE max [lm/W]	At 1000 nit[%, V]
2:1	2.3	62920 (9.8 V)	3608	13.7%, 32.1	38.8	12.5%, 3.4
1:1	2.3	77120 (10.0 V)	4152	16.0%, 37.8	47.5	14.7 %, 3.4
1:2	2.3	109560 (9.8 V)	4665	14.9%,	44.6	14.1 %, 3.4

Supplementary Table 3. Vibration peaks and lifetimes of CN-Cz2, PO-T2T and CN-Cz2:PO-T2T in Step-Scan spectra.

CN-Cz2		PO-T2T		CN-Cz2:PO-T2T	
IR (cm^{-1})	τ (μs)	IR (cm^{-1})	τ (μs)	IR (cm^{-1})	τ (μs)
				1192	3.25
				1319	3.60
1477	7.15			1473	3.08 (-), 15.21
				1489	3.11 (-), 15.12
		1512	5.02	1508	14.89
		1527	5.07 (-)	1525	14.52 (-)
				1547	3.56
		1562	5.08	1558	3.60
				1570	3.12
1597	7.12			1597	15.12 (-)
				2060	3.08
2214	7.47			2214	14.79
2221	7.01			2221	3.08, 14.80 (-)

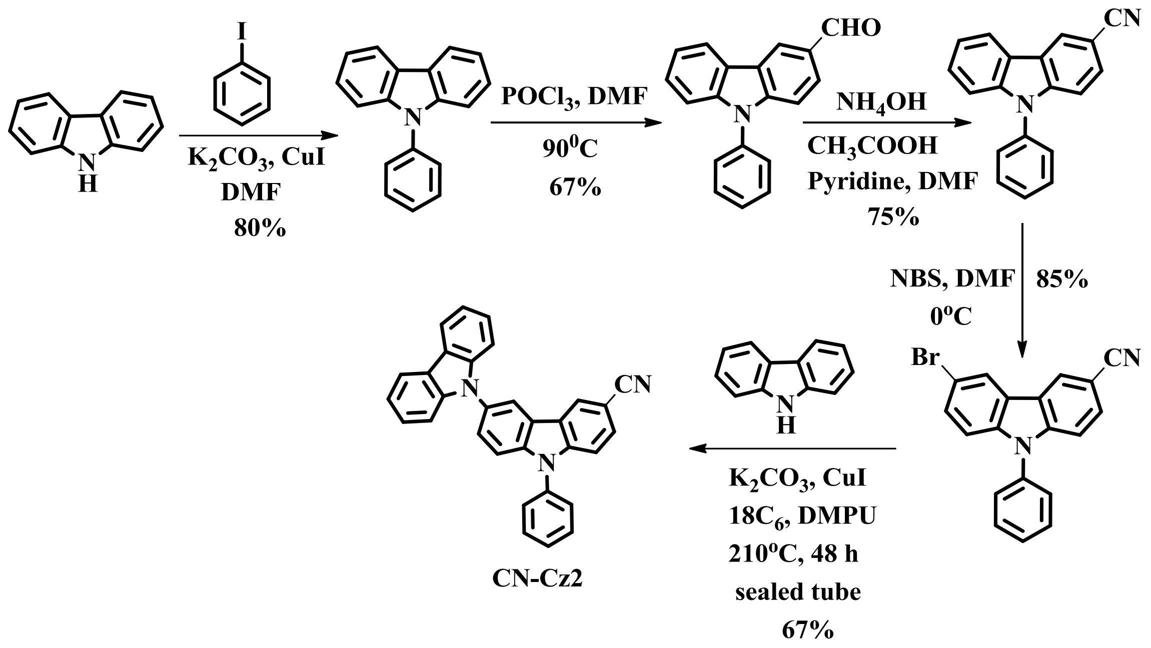

Supplementary Figure 1. Synthesis of CN-Cz2

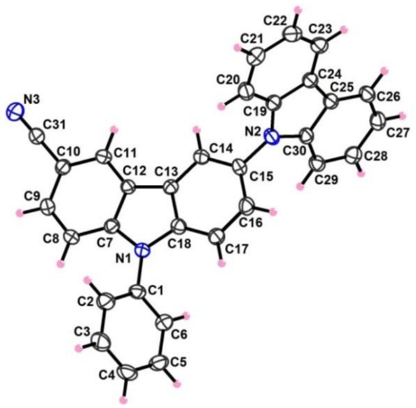

Supplementary Figure 2. Thermal ellipsoidal plot of CN-Cz2 (50% probability).

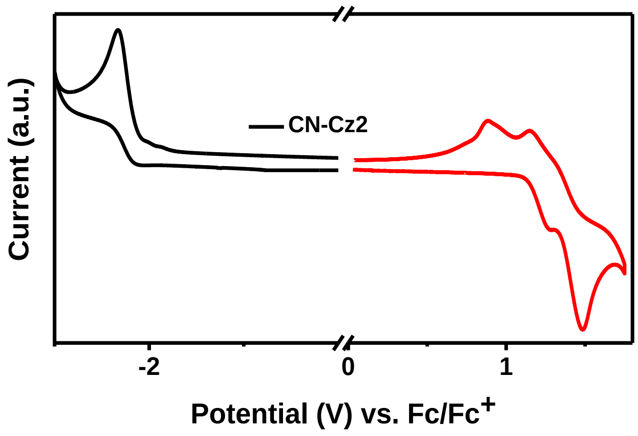

Supplementary Figure 3. Cyclic voltammograms of CN-Cz2. The measurement of oxidation potential was performed in CH_2Cl_2 with 0.1 M of *n*Bu_4NPF_6 as a supporting electrolyte, whereas the reduction CV was performed in DMF with 0.1 M of *n*Bu_4NClO_4 as a supporting electrolyte at a scan rate of 100 mV s^{-1}.

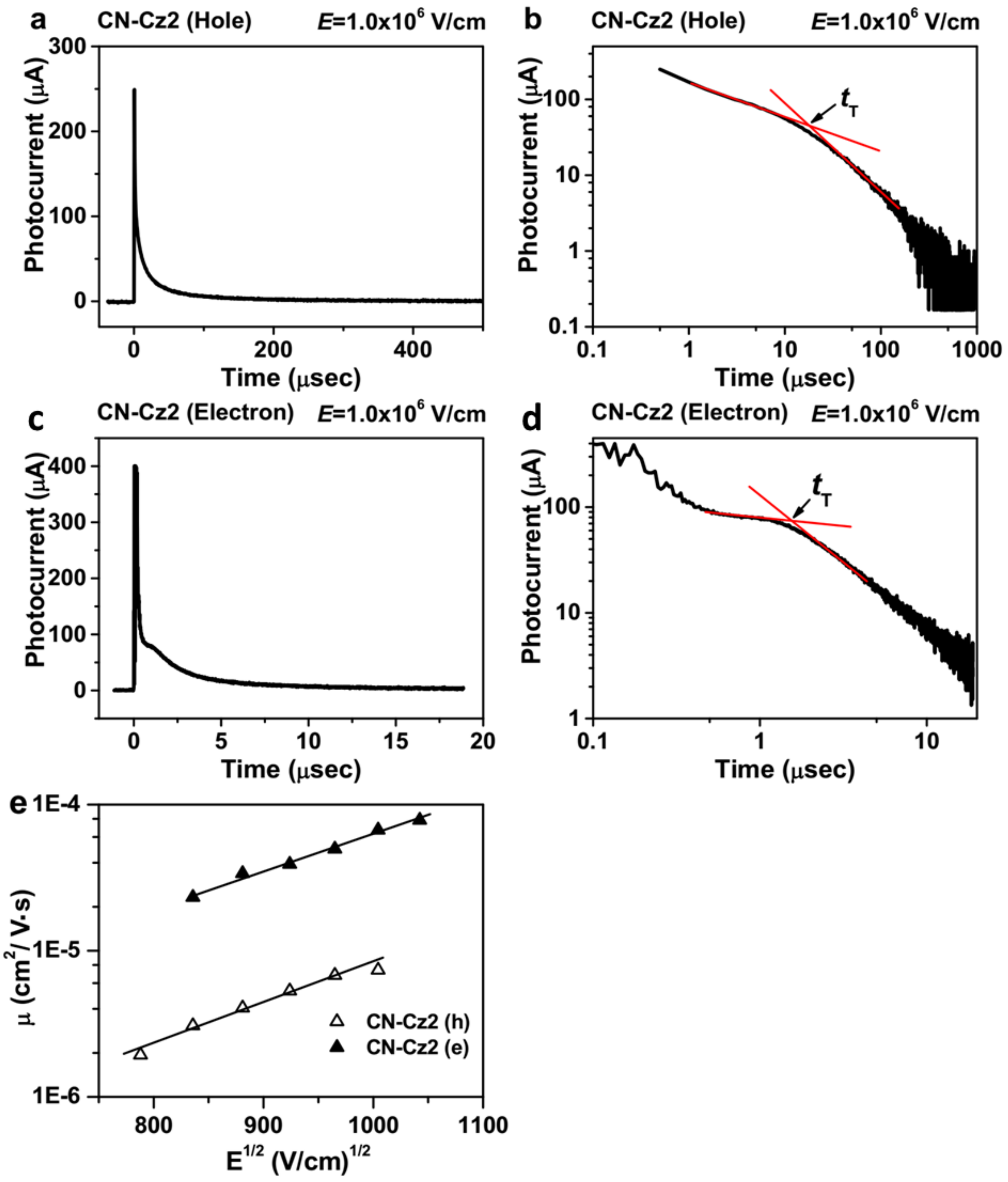

Supplementary Figure 4. Representative TOF transients of CN-Cz2. **a.** holes (linear), **b.** holes (log10), **c**. electrons (linear) and **d**. electrons (log10) at $E = 1.0 \times 10^6$ V/cm. **e.** Carrier mobility of CN-Cz2 plotted with respect to $E^{1/2}$.

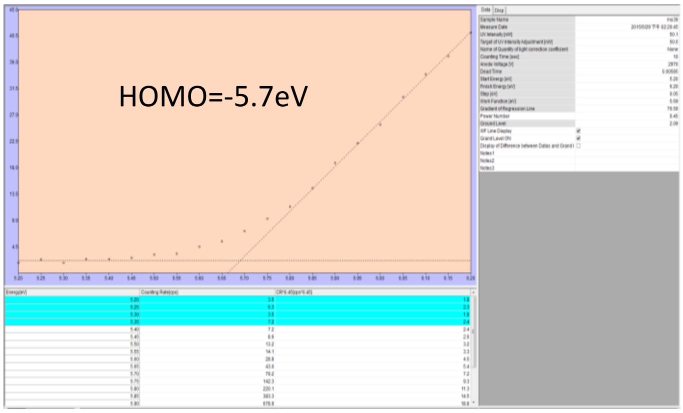

Supplementary Figure 5. HOMO energy level of CN-Cz2 determined by using photoelectron yield spectroscopy (Riken AC-2).

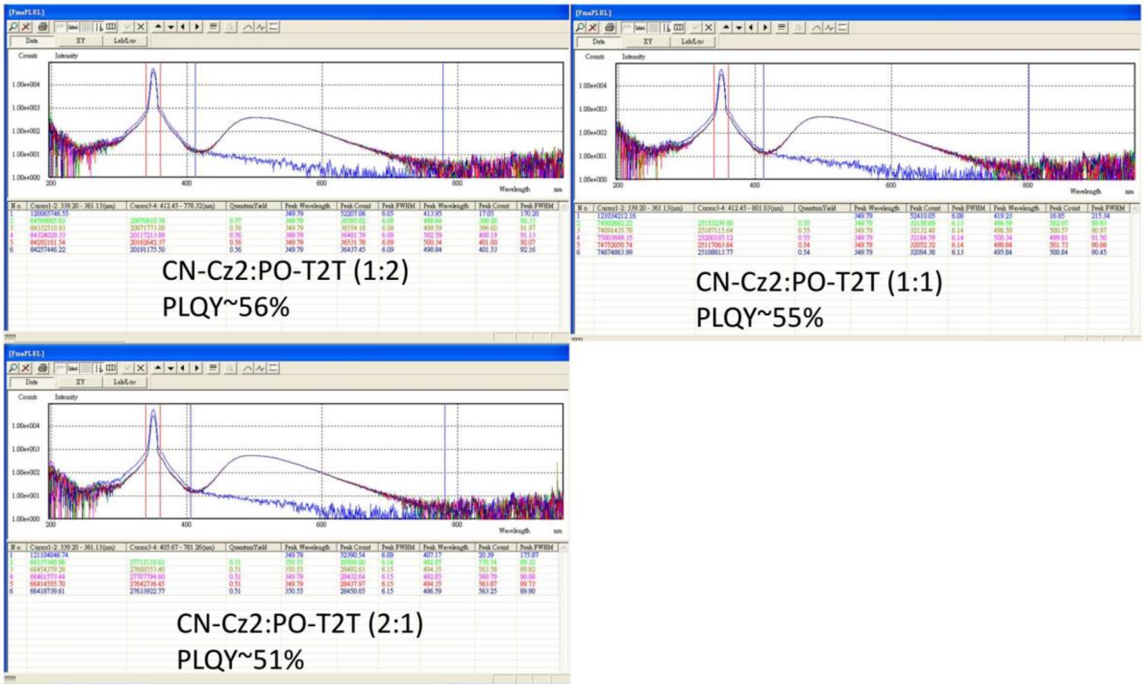

Supplementary Figure 6. PLQY for CN-Cz2:PO-T2T with different weight ratio. Quantum efficiency measurements were recorded with an integration sphere coupled with a photonic multi-channel analyzer (Hamamatsu C9920).

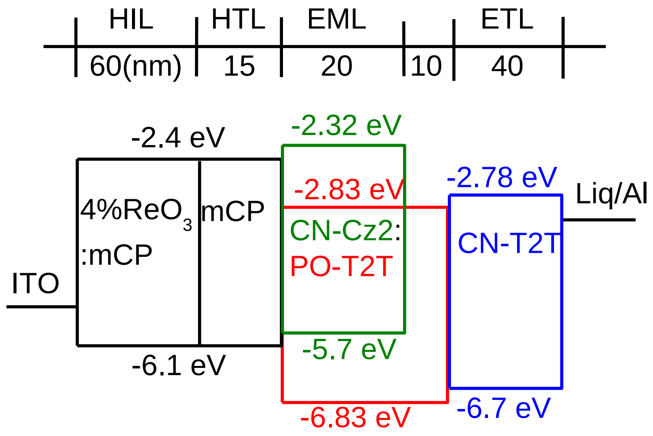

Supplementary Figure 7. Device structure of the CN-Cz2:PO-T2T exciplex OLED. ITO/4% ReO_3: mCP (60 nm)/ mCP (15 nm)/ CN-Cz2:PO-T2T (1:1 weight ratio) (20 nm)/ PO-T2T (10 nm)/CN-T2T (40 nm)/Liq (0.5 nm)/Al (100 nm).

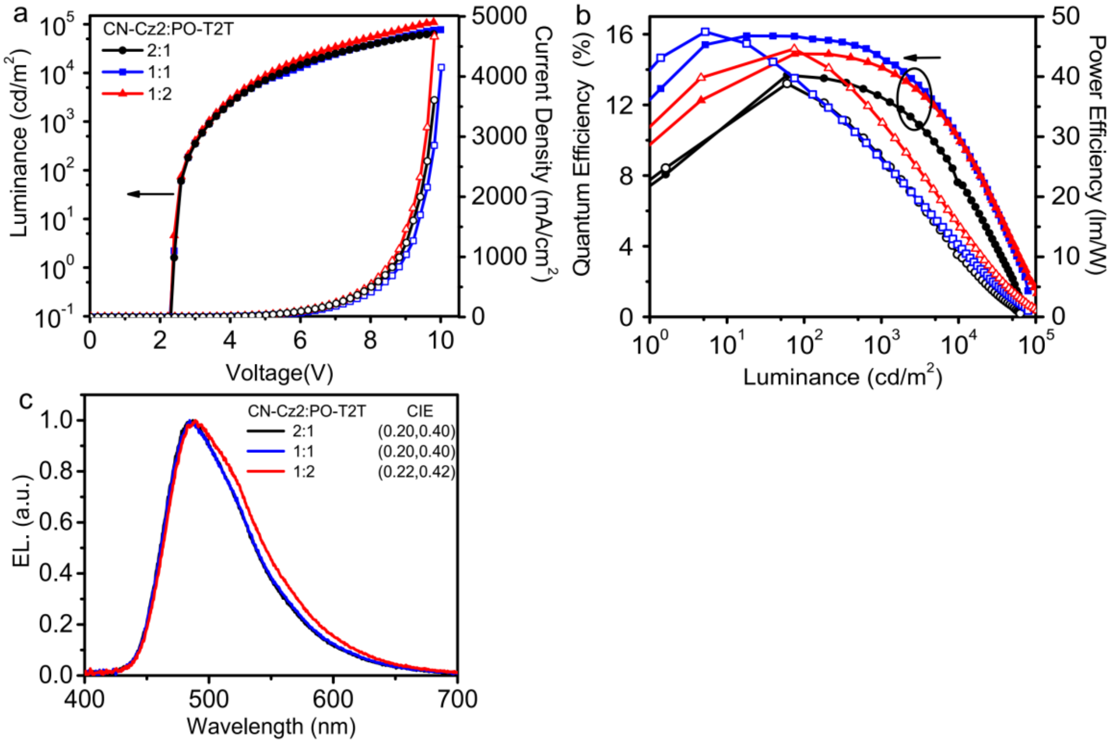

Supplementary Figure 8. The performance parameters of OLEDs. **a** *J–V–L* characteristics, **b** *EQE–PE–L* characteristics, **c** Normalized EL spectra for CN-Cz2:PO-T2T with different weight ratio.

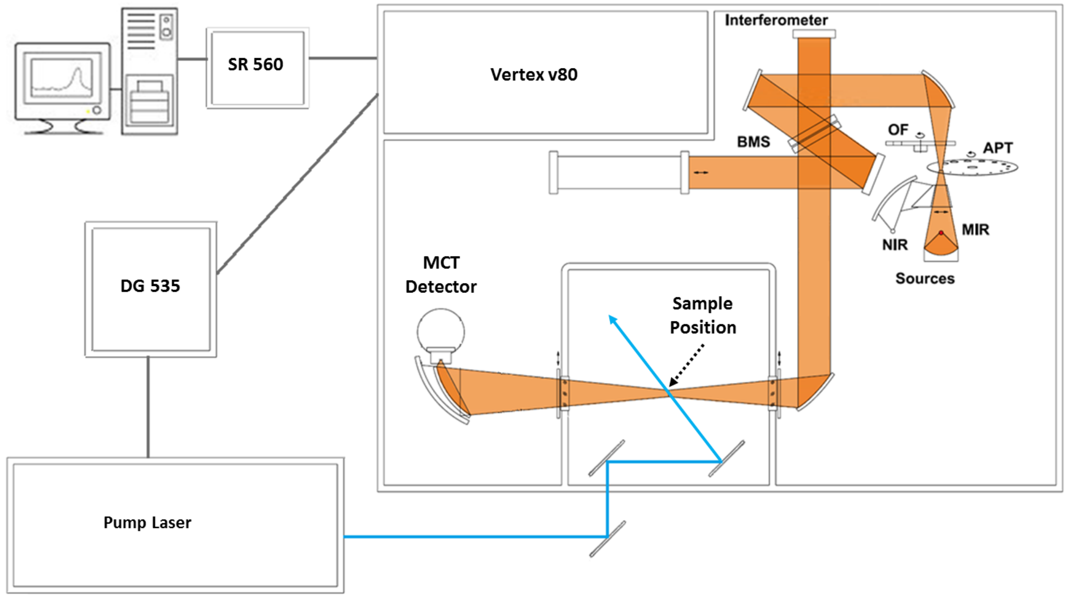

Supplementary Figure 9. The setup of Step-Scan FTIR experiment. NIR: near infrared, MIR: middle infrared BMS: beam splitter, APT: aperture, OF: optical filter, DG 535: the electronic triggering system, SR 560: voltage preamplifier.

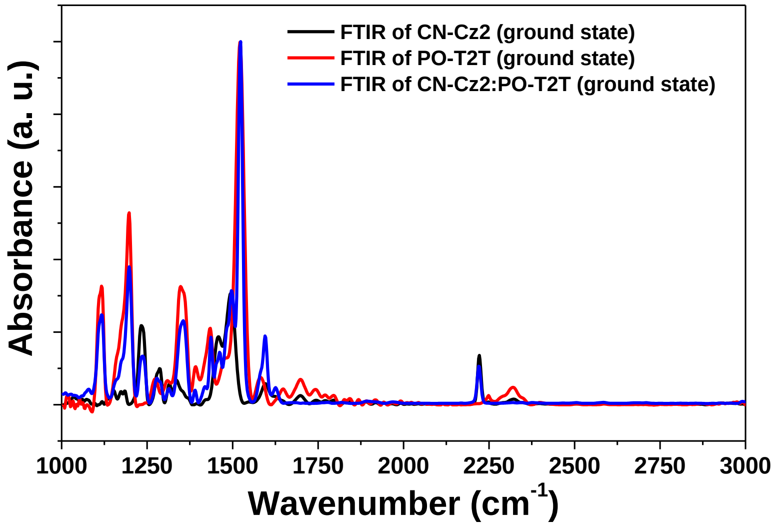

Supplementary Figure 10. The steady-state IR spectra of CN-Cz2, PO-T2T and CN-Cz2:PO-T2T.

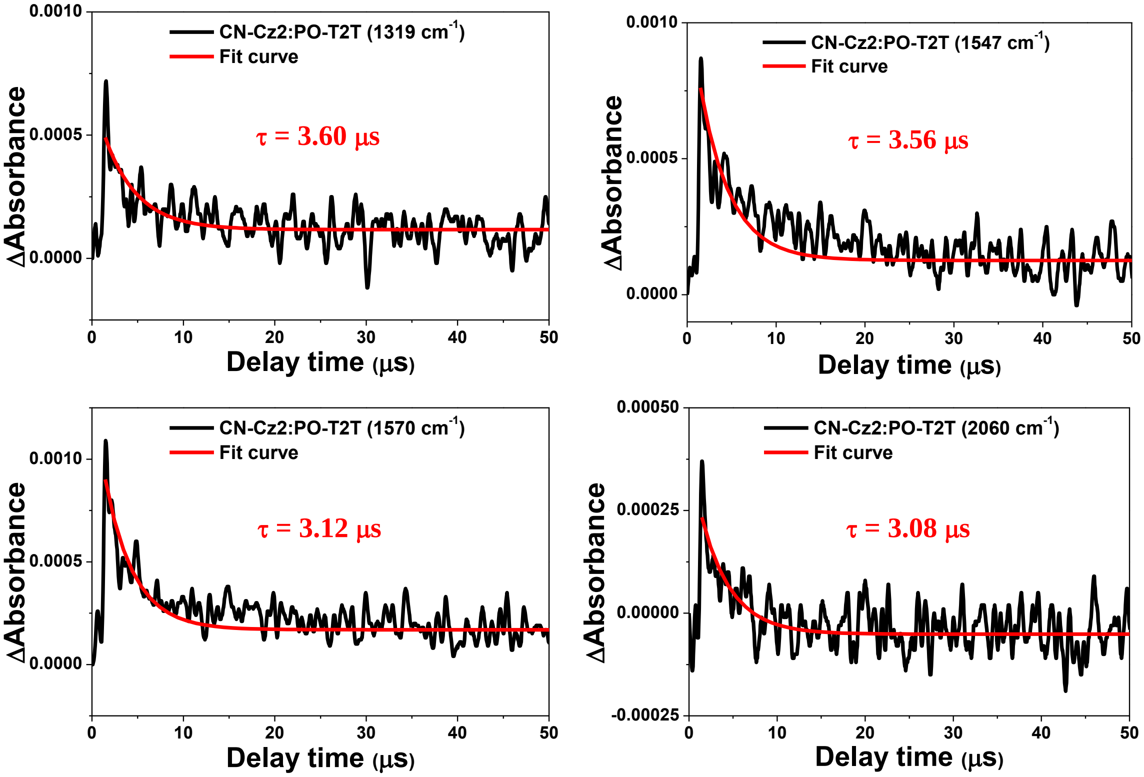

Supplementary Figure 11. The transient IR character. The decay profiles of new generated exciplex band (1319, 1547, 1570 cm^{-1} and band ~2060 cm^{-1}) of CN-Cz2:PO-T2T exciplex.

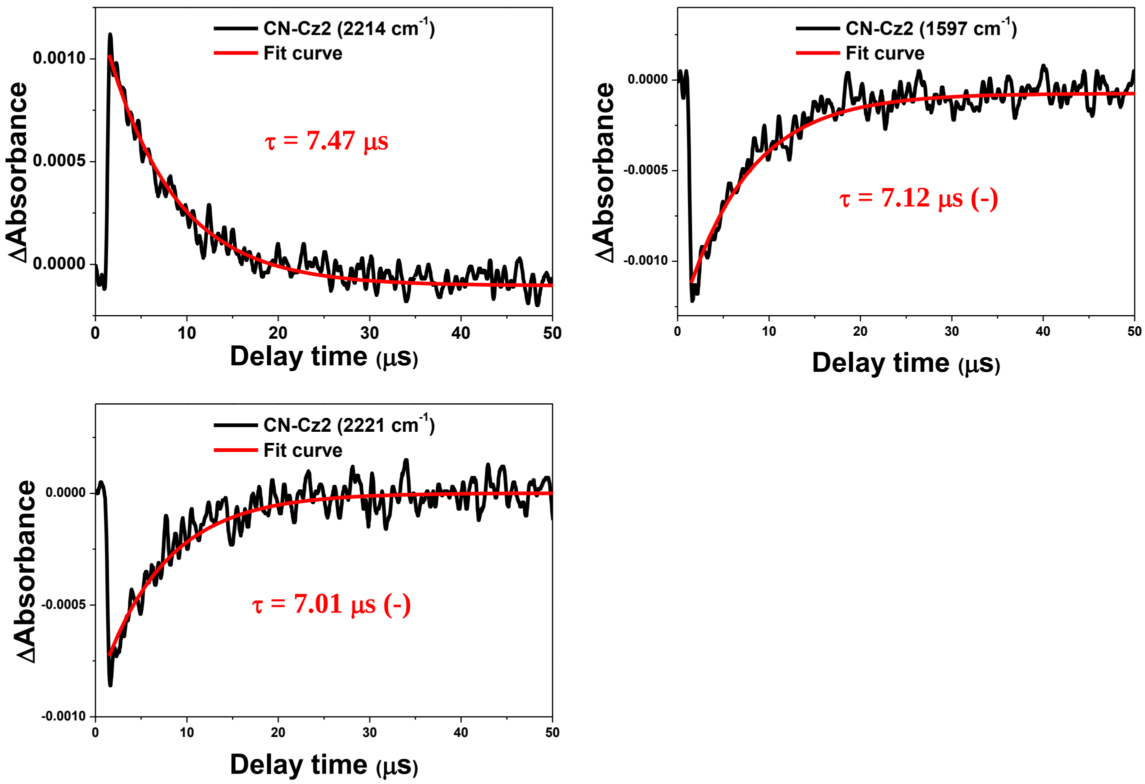

Supplementary Figure 12. The transient IR character. The decay (2214 cm^{-1} peak) and rise (1597, 2221 cm^{-1} peak) dynamics of CN-Cz2.

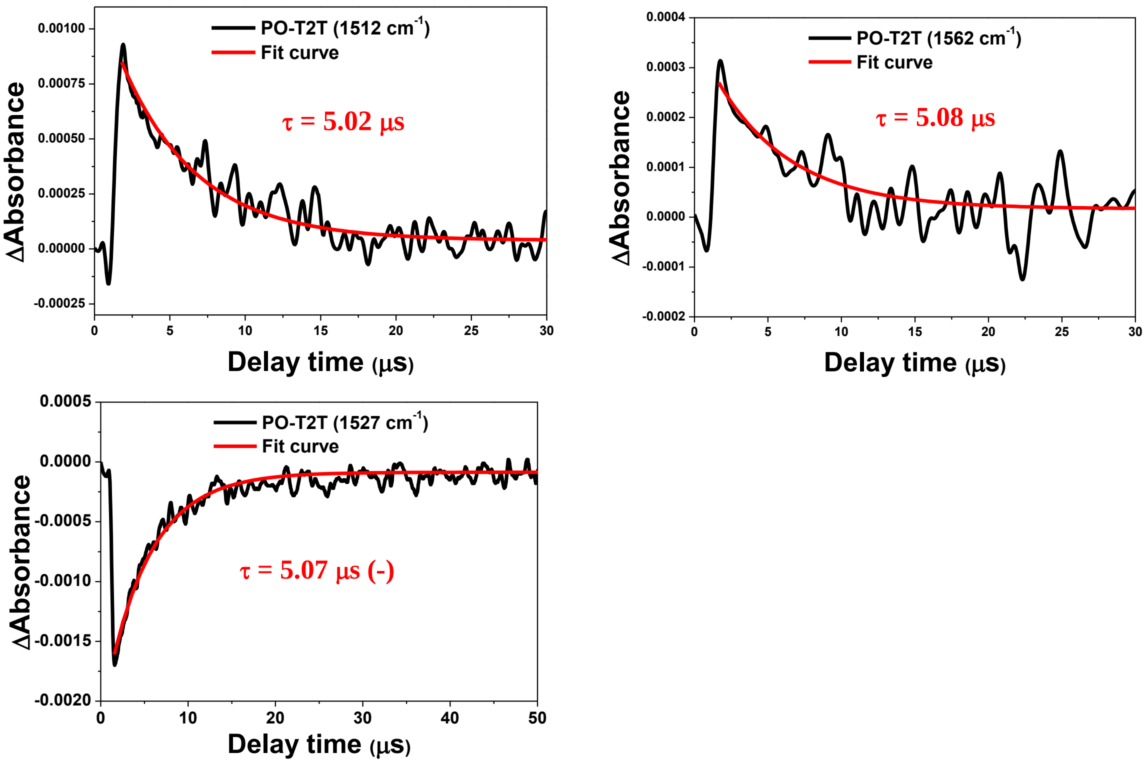

Supplementary Figure 13. The transient IR character. The decay (1512, 1562 cm^{-1} peak) and rise (1527 cm^{-1} peak) dynamics of PO-T2T.

a.

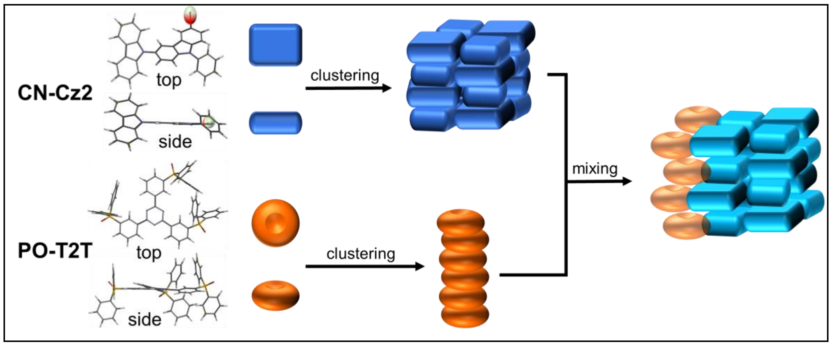

b.

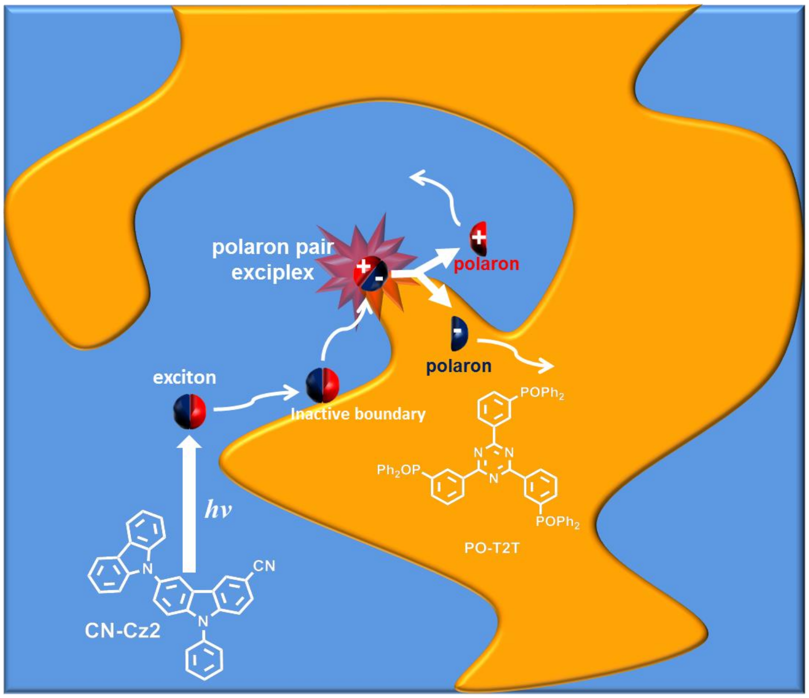

Supplementary Figure 14. The structure of molecular packing and polaron formation. (a) CN-Cz2 grain domain, PO-T2T grain domain, and the in-plane interaction between CN-Cz2 and PO-T2T grain domains. Note that the 1D GIXD profile of PO-T2T shows a strong diffraction peak at $2\vartheta = 15.5^{o}$, corresponding to a well correlated π-π stacking with a d-spacing of 3.8 Å (see Figure 3 and relevant results in text for detail). (b) The illustration of exciton, exciplex and polaron generation in the mixture of CN-Cz2:PO-T2T (1:1) co-deposited by vapor evaporation. For clarity the packing of CN-Cz2 and PO-T2T is simply drawn in homogenous blue and orange colors, respectively. In this illustration the excitation starts from CN-Cz2. Note that the excitons remains intact when they

encounter an inactive boundary where CN-Cz2 and PO-T2T do not interact. Until they encounter an active interface where CN-Cz2 and PO-T2T interact, the charge transfer takes place, forming exciplex.

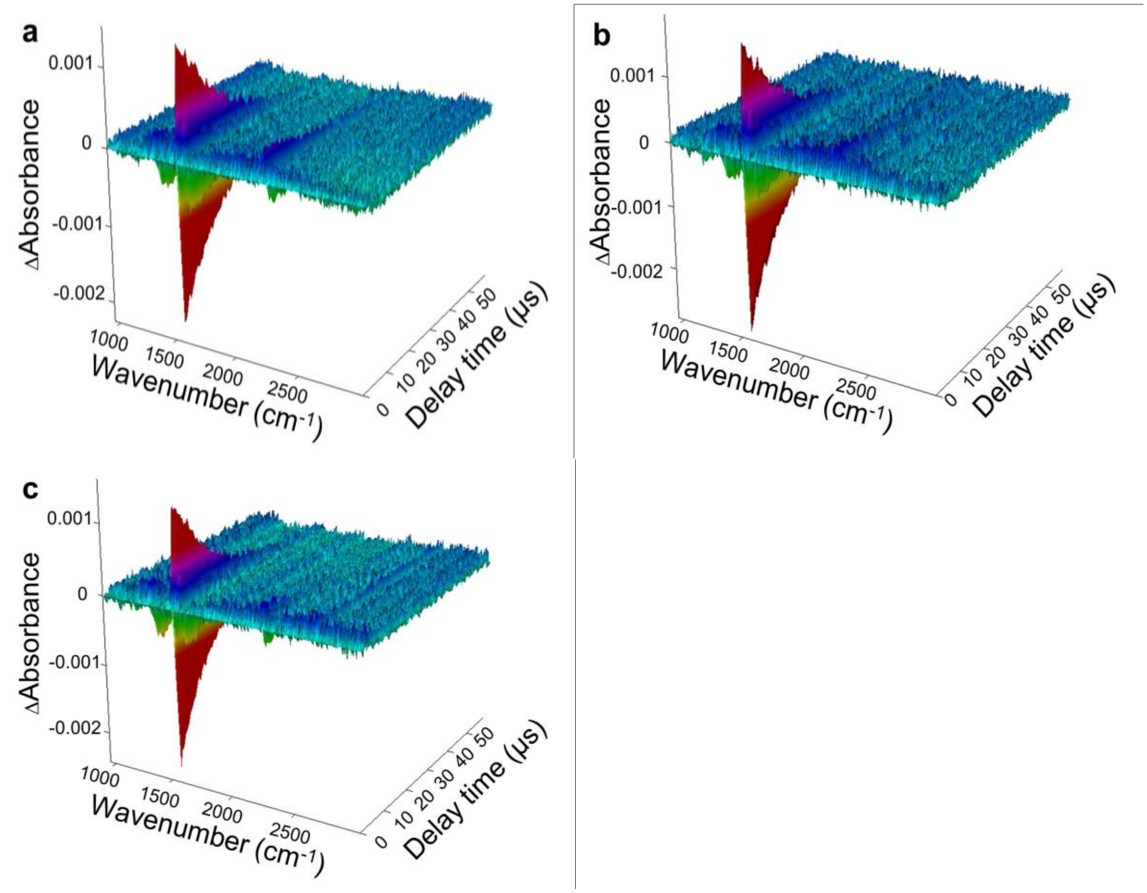

Supplementary Figure 15. The transient IR character. Absorbance-frequency-time 3D plot for the CN-Cz2:PO-T2T with different weight ratio **a.**(2:1), **b.** (1:1) and **c.** (1:2).

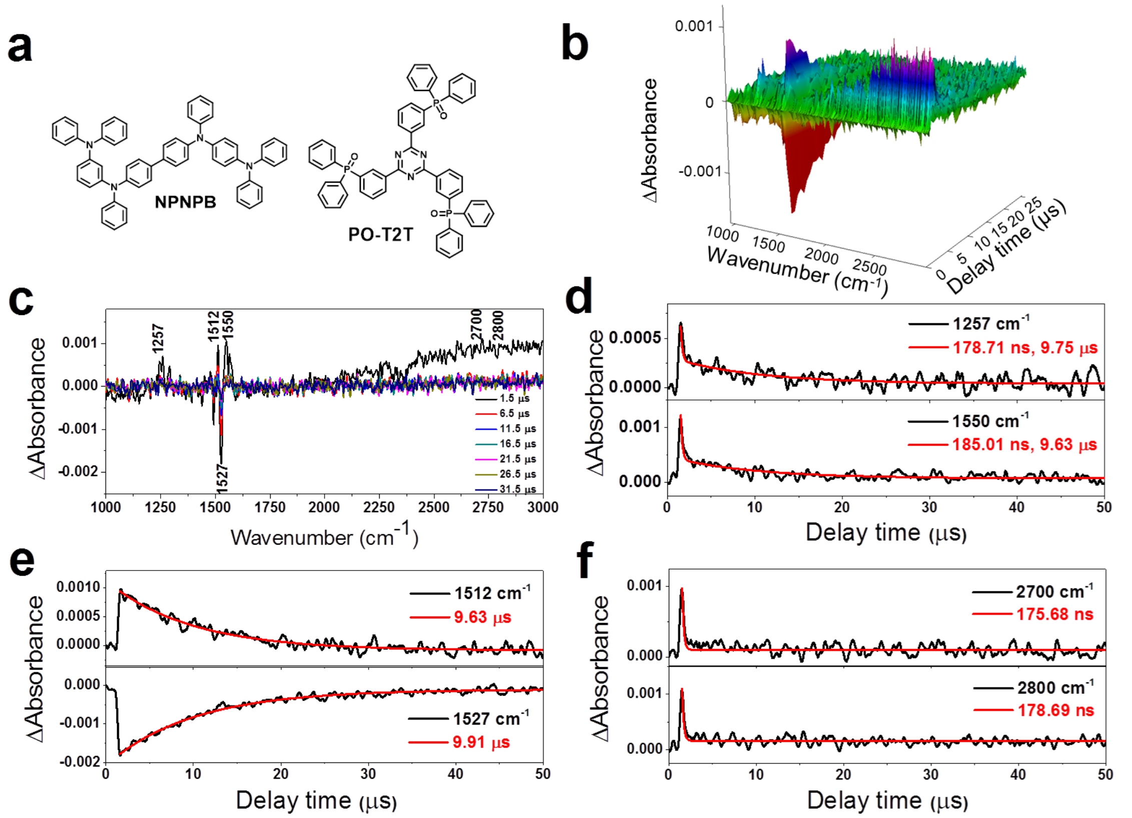

Supplementary Figure 16. The transient IR character of exciplex composed of NPNPB and PO-T2T. **a.** Chemical structures of NPNPB and PO-T2T. **b.** An absorbance-frequency-time 3D plot for the NPNPB:PO-T2T (1:1). Excitation wavelength: 266 nm. **c.** The transient IR spectra of NPNPB:PO-T2T film at different delay times in spectral range of 1000-3000 cm^{-1}. **d.** The relaxation dynamics of 1257 and 1550 cm^{-1}, **e.** 1512 and 1527 cm^{-1} and **f.** 2700 and 2800 cm^{-1} polaron-pair absorption band for the NPNPB:PO-T2T film. The PL decay lifetime is about 180 ns. NPNPB: *N,N′*-diphenyl-*N,N′*-di-[4-(*N,N*-diphenyl-amino)phenyl]benzidine

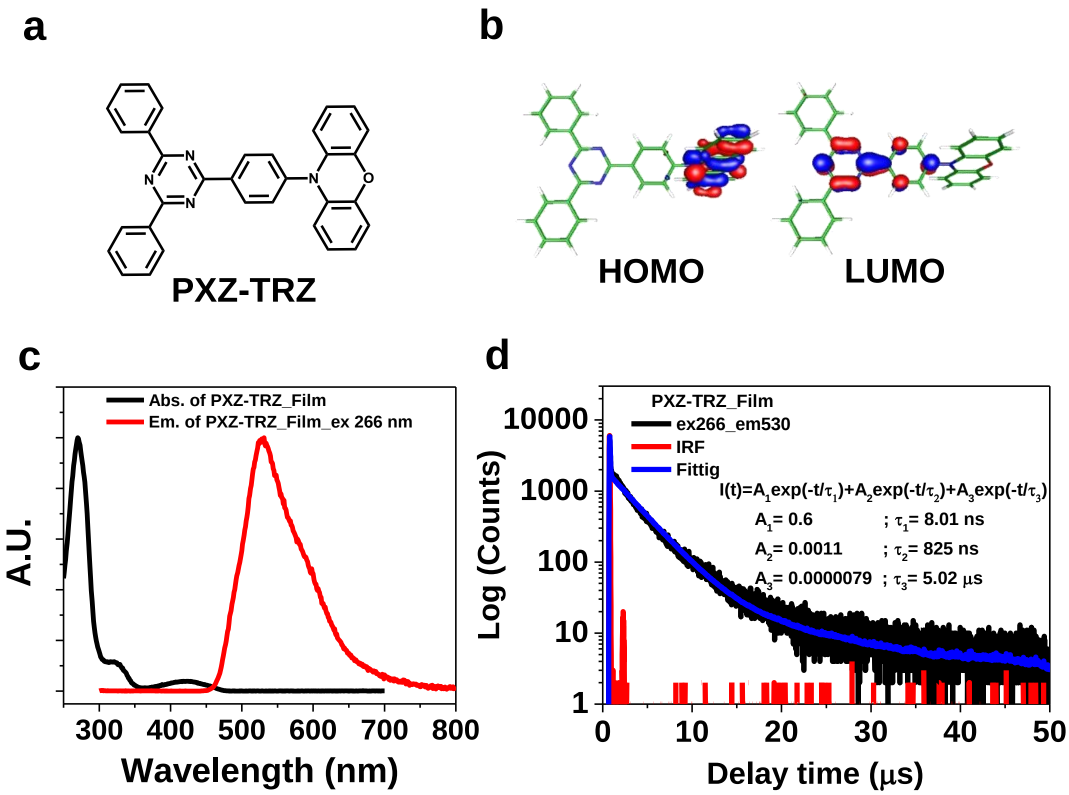

Supplementary Figure 17. Photophysical property of PXZ-TRZ. **a.** Chemical structure of PXZ-TRZ. **b.** HOMO and LUMO orbitals of PXZ-TRZ according to DFT calculations at the B3LYP/6-31G(d) level. **c.** Normalized photoluminescence (PL) and absorption spectra of PXZ-TRZ. **d.** Transient PL decay properties of PXZ-TRZ blend film at 300 K. PXZ-TRZ: 10-(4-(4,6-Diphenyl-1,3,5-triazin-2-yl)phenyl)-10H-phenoxazine.

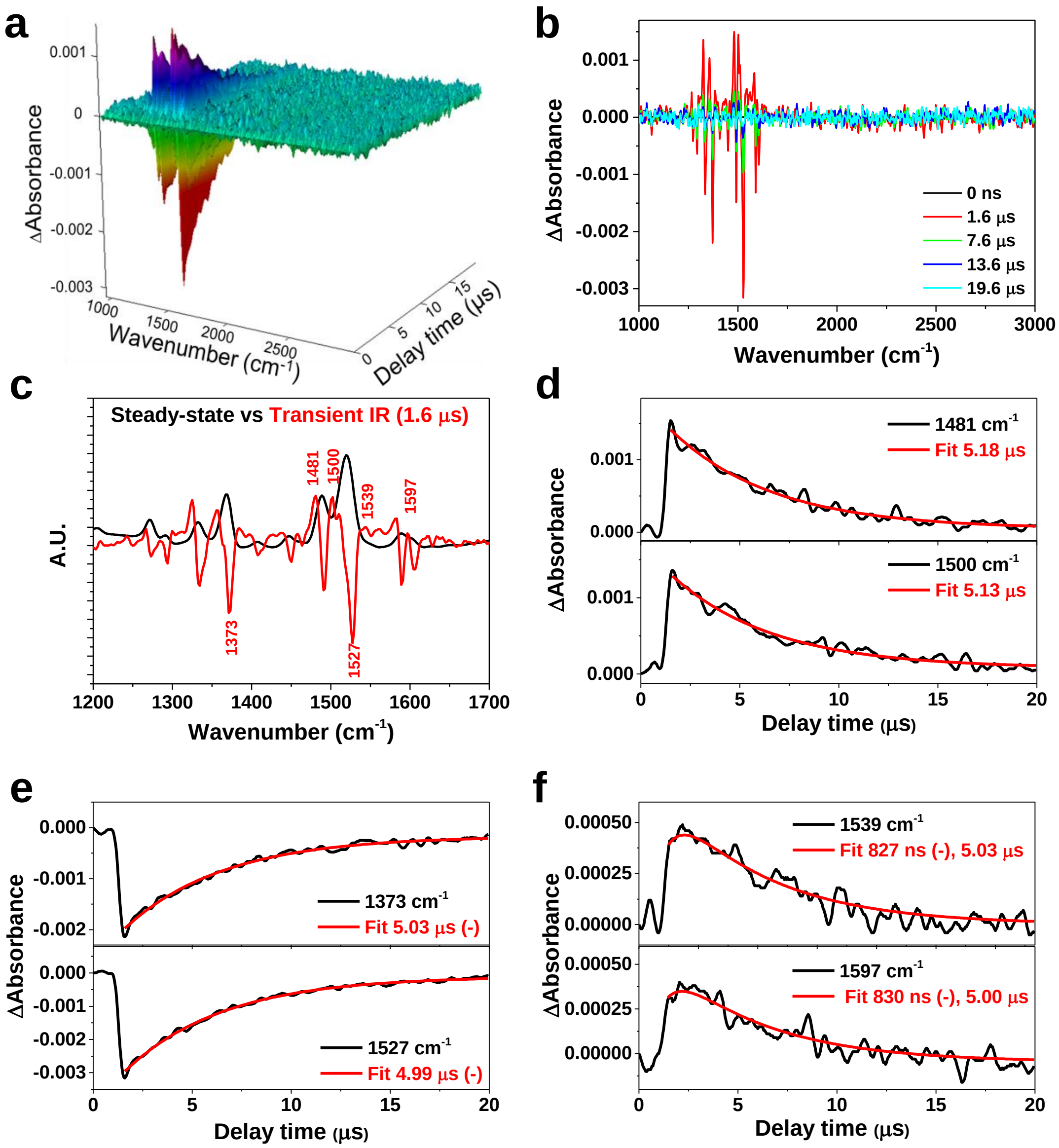

Supplementary Figure 18. The transient IR character of PXZ-TRZ. a. An absorbance-frequency-time 3D plot for the transient IR of PXZ-TRZ film. Excitation wavelength: 266 nm. **b.** The transient IR spectraof PXZ-TRZ at different delay times in spectral range of 1000-3000 cm^{-1}. **c.** Comparison spectra of steady-state IR absorption and transient IR at 1.6 μs delayed time. **d.** The relaxation dynamics of 1481 and 1500 cm^{-1}, **e.** 1373 and 1527 cm^{-1} and **f.** 1539 and 1597 cm^{-1} peaks for the PXZ-TRZ. PXZ-TRZ: 10-(4-(4,6-Diphenyl-1,3,5-triazin-2-yl)phenyl)-10H-phenoxazine.

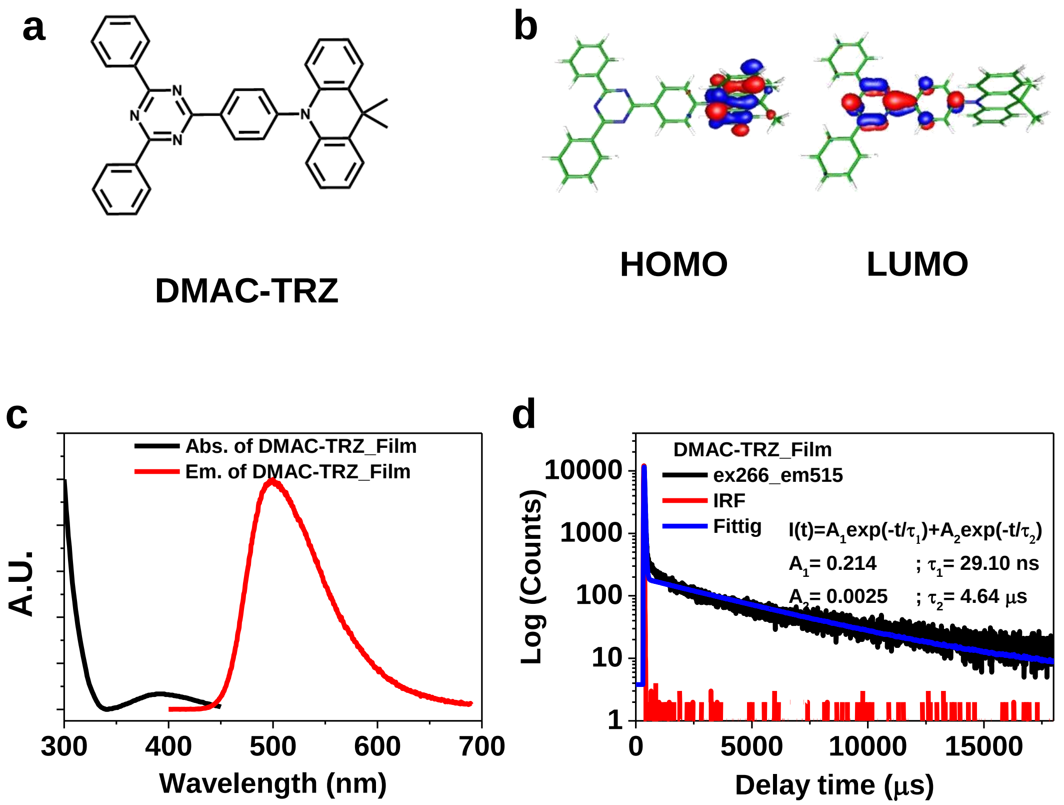

Supplementary Figure 19. Photophysical property of DMAC-TRZ. **a.** Chemical structure of DMAC-TRZ. **b.** HOMO and LUMO orbitals of DMAC-TRZ according to DFT calculations at the B3LYP/6-31G(d) level. **c.** Normalized photoluminescence (PL) and absorption spectra of DMAC-TRZ. **d.** Transient PL decay properties of DMAC-TRZ blend film at 300 K. DMAC-TRZ: 9,10-Dihydro-9,9-dimethyl-10-(4-(4,6-diphenyl-1,3,5 -triazin-2-yl)phenyl)acridine.

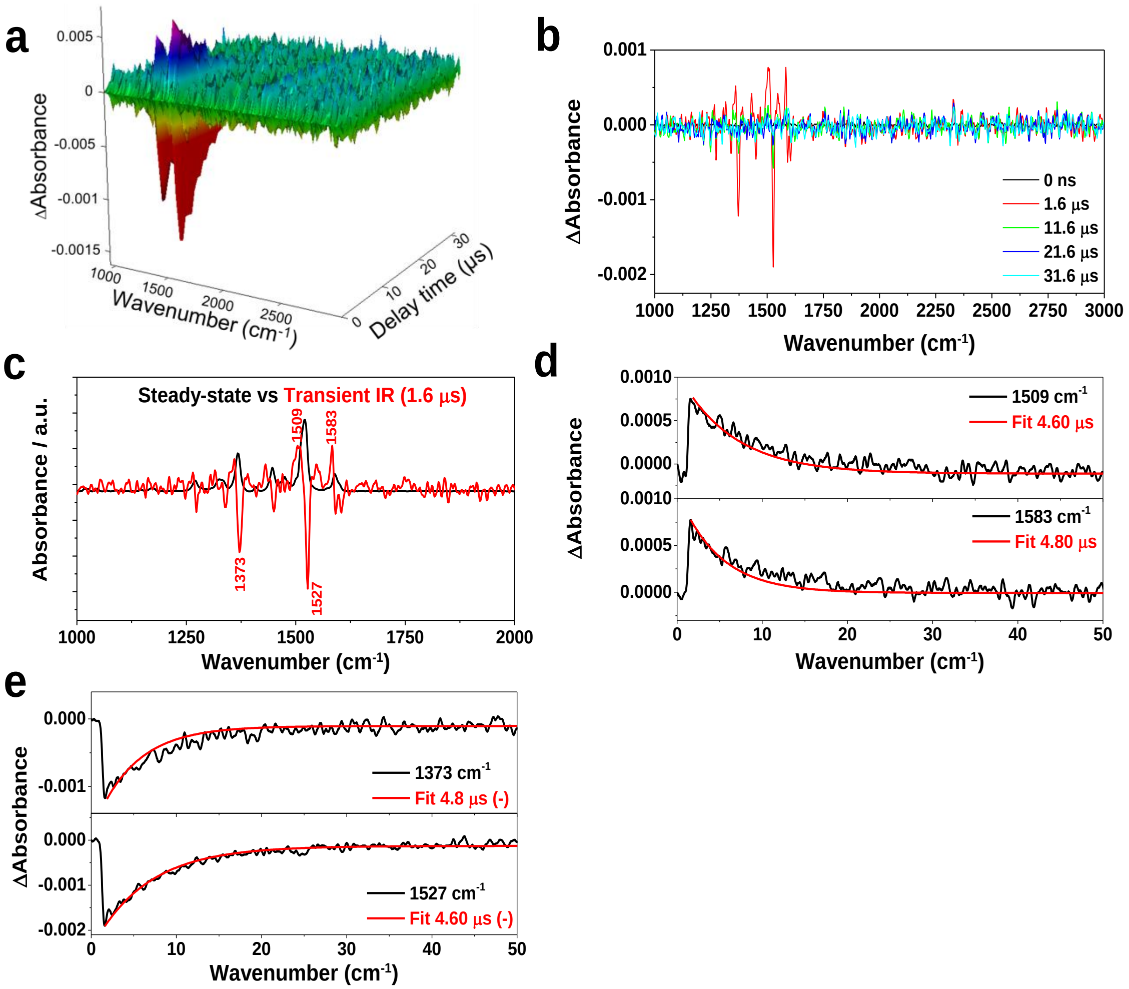

Supplementary Figure 20. The transient IR character of DMAC-TRZ. **a.** An absorbance-frequency-time 3D plot for the transient IR of DMAC-TRZ film. Excitation wavelength: 266 nm. **b.** The transient IR spectraof DMAC-TRZ at different delay times in spectral range of 1000-3000 cm^{-1}. **c.** Comparison spectra of steady-state IR absorption and transient IR at 1.6 μs delayed time. **d.** The relaxation dynamics of 1509 and 1583 cm^{-1}, **e.** 1373 and 1527 cm^{-1} peaks for the DMAC-TRZ. DMAC-TRZ: 9,10-Dihydro-9,9-dimethyl-10-(4-(4,6-diphenyl-1,3,5 -triazin-2-yl)phenyl)acridine.

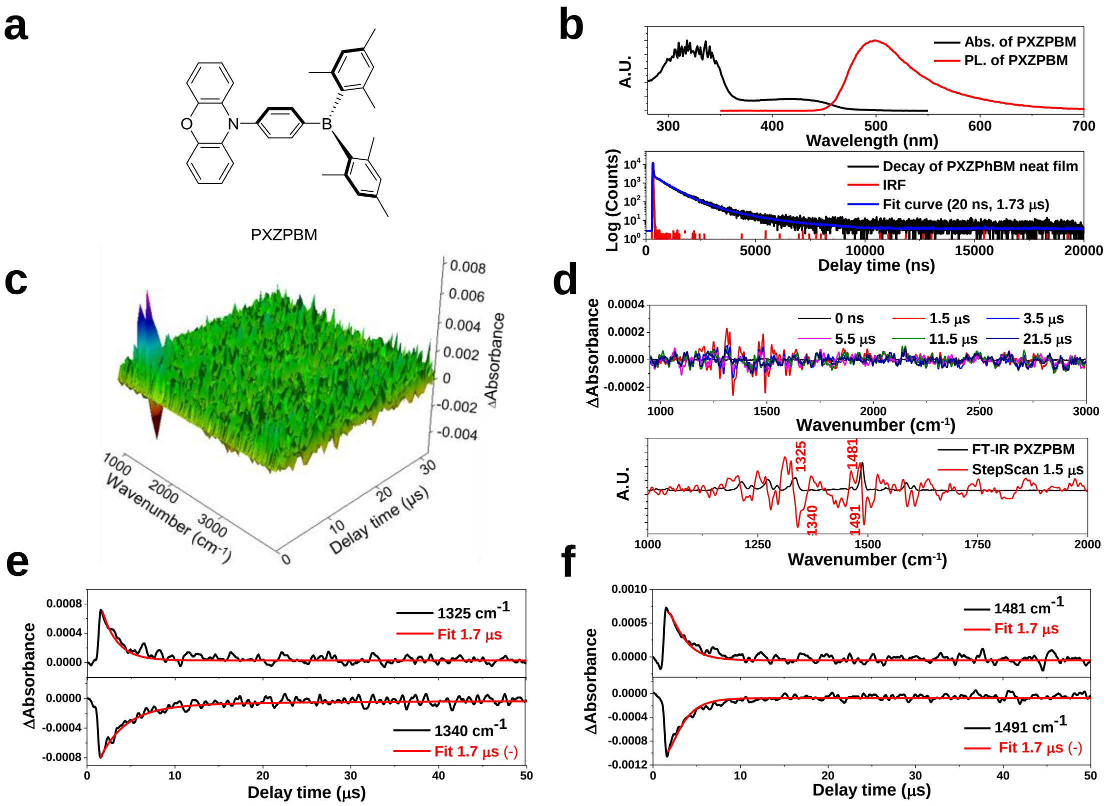

Supplementary Figure 21. Photophysical property and the transient IR character of PXZPBM. **a.** Chemical structure of PXZPBM.[6] **b.** Normalized photoluminescence (PL), absorption spectra and decay of PXZPBM neat film. **c.** An absorbance-frequency-time 3D plot for the transient IR of PXZPBM film. Excitation wavelength: 266 nm. **d.** The transient IR spectra of PXZPBM at different delay times and comparison spectra of steady-state IR absorption and transient IR at 1.5 μs delayed time. **e.** 1325 and 1340 cm^{-1} peaks for the PXZPBM. **f.** 1481 and 1491 cm^{-1} peaks for the PXZPBM. PXZPBM: 10-(4-dimesitylborylphenyl)phenoxazine.

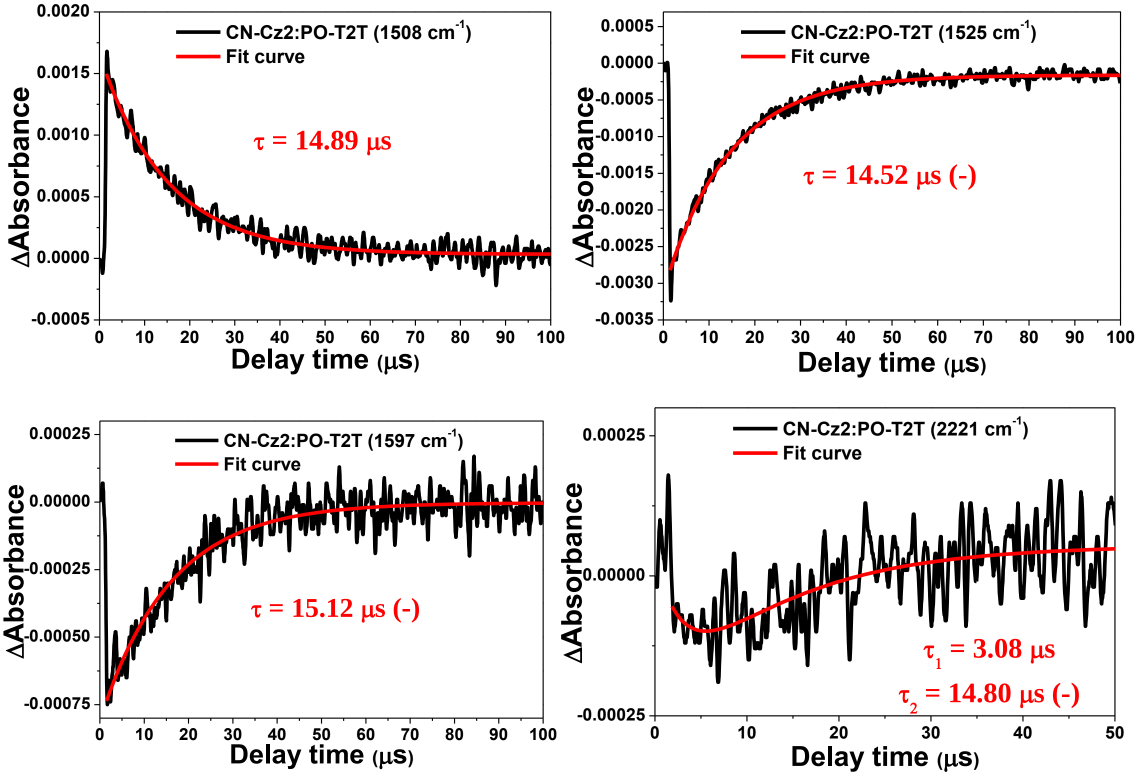

Supplementary Figure 22. The transient IR character. The decay 1508 cm^{-1} and rise (1525, 1597, 2221 cm^{-1}) dynamics of CN-Cz2:PO-T2T exciplex.

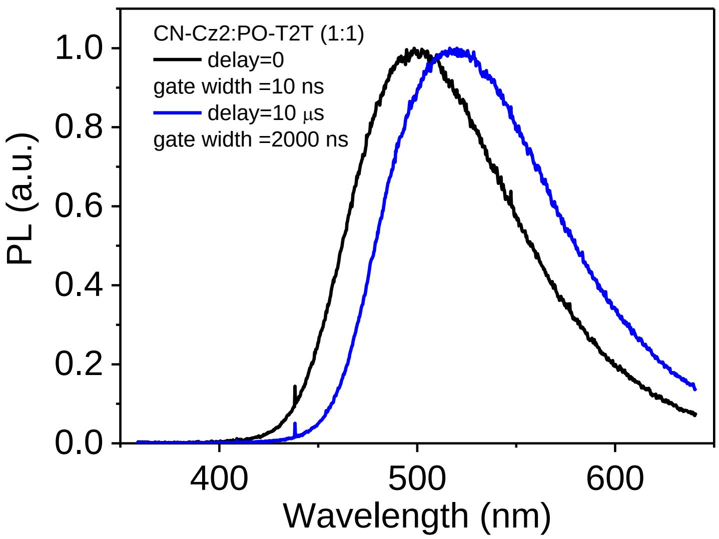

Supplementary Figure 23. Transient PL decay properties of CN-Cz2:PO-T2T film. Prompt (delay = 0, gate width = 10 ns) and delayed (delay = 10 μs, gate width = 2 μs) components of PL spectra for the CN-Cz2:PO-T2T film at 300 K. A slight red shift was resolved between the prompt and delayed components.

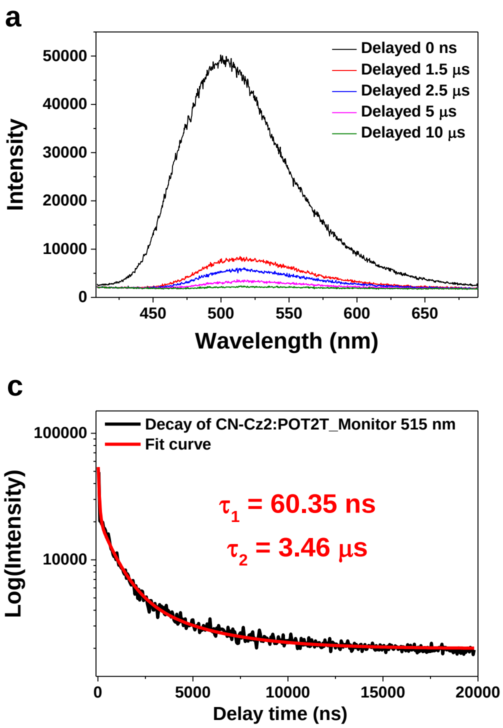

Supplementary Figure 24. Transient EL decay properties of CN-Cz2:PO-T2T film. **a.** Transient Electroluminescence (EL) spectra of CN-Cz2:PO-T2T at different delayed times. **b.** Normalized EL spectra of CN-Cz2:PO-T2T at different delayed times. **c.** Decay of EL of CN-Cz2:PO-T2T at 515 nm.

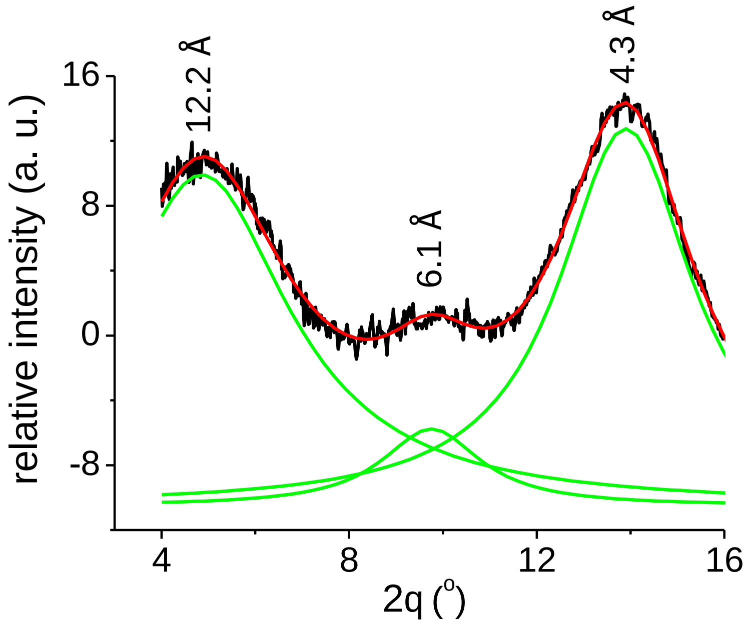

Supplementary Figure 25. The GIXD character. The fitted 1D GIXD of CN-Cz2 film.

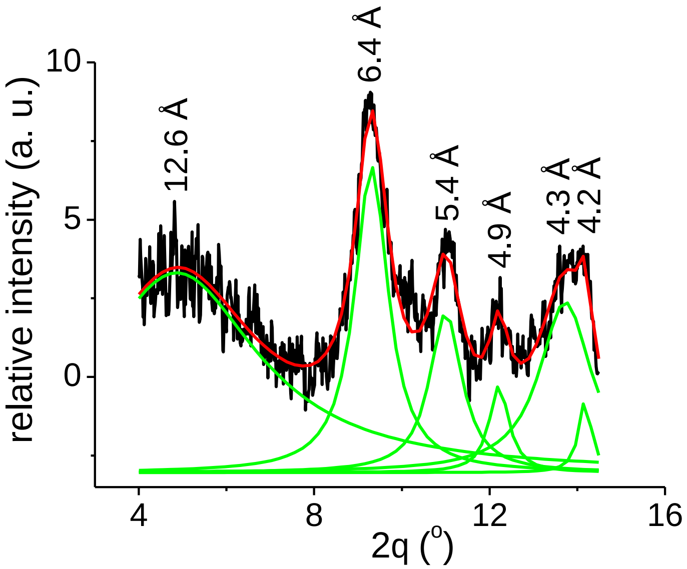

Supplementary Figure 26. The GIXD character. Fitted 1D GIXD of co-deposited film.

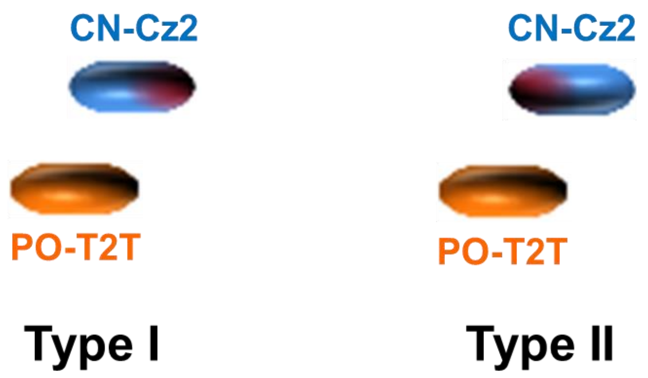

Supplementary Figure 27. The arrangement of CN-Cz2 and PO-T2T to form the exciplex. These arrangements are drawn according to the results of GIXD, in which we assume that CN-Cz2 and PO-T2T approach in an in-plane direction. As a result, the –CN ligand (red region) of CN-Cz2 can be in two orientations, far away from or toward PO-T2T.

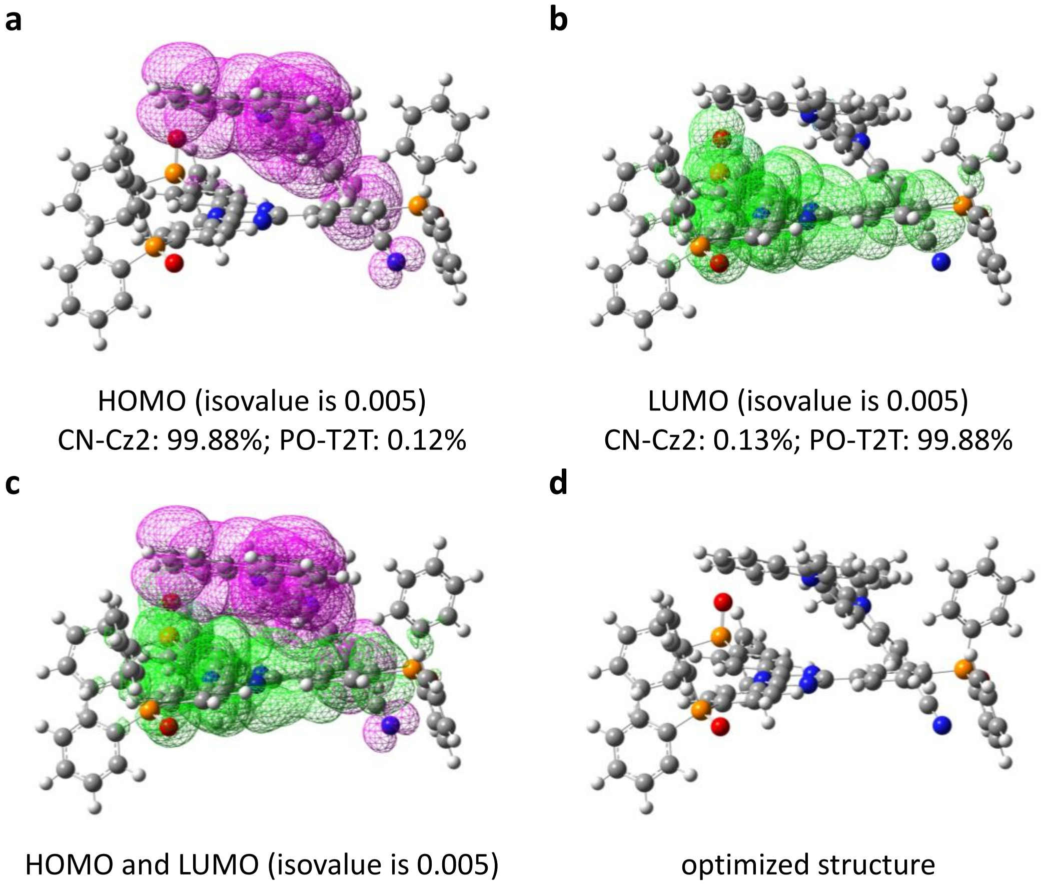

Supplementary Figure 28. Theoretical calculations based on the type I arrangement of CN-Cz2 and PO-T2T. The optimized structure of S_1 for the type I of CN-Cz2:PO-T2T exciplex in dichloromethane. The pink and green mesh are standing for (a) HOMO and (b) LUMO, respectively. The isovalue 0.02 for the coutours is defalut in GaussView. When the isolvalue is smaller (0.005), the electron density distribution "vision" for the orbital is larger. (c) The electron density distributions of HOMO and LUMO are showed. (d) The distance between the carbazole of CN-Cz2 and 1,3,5-triazine of PO-T2T is estimated around 4.02 Å**.**

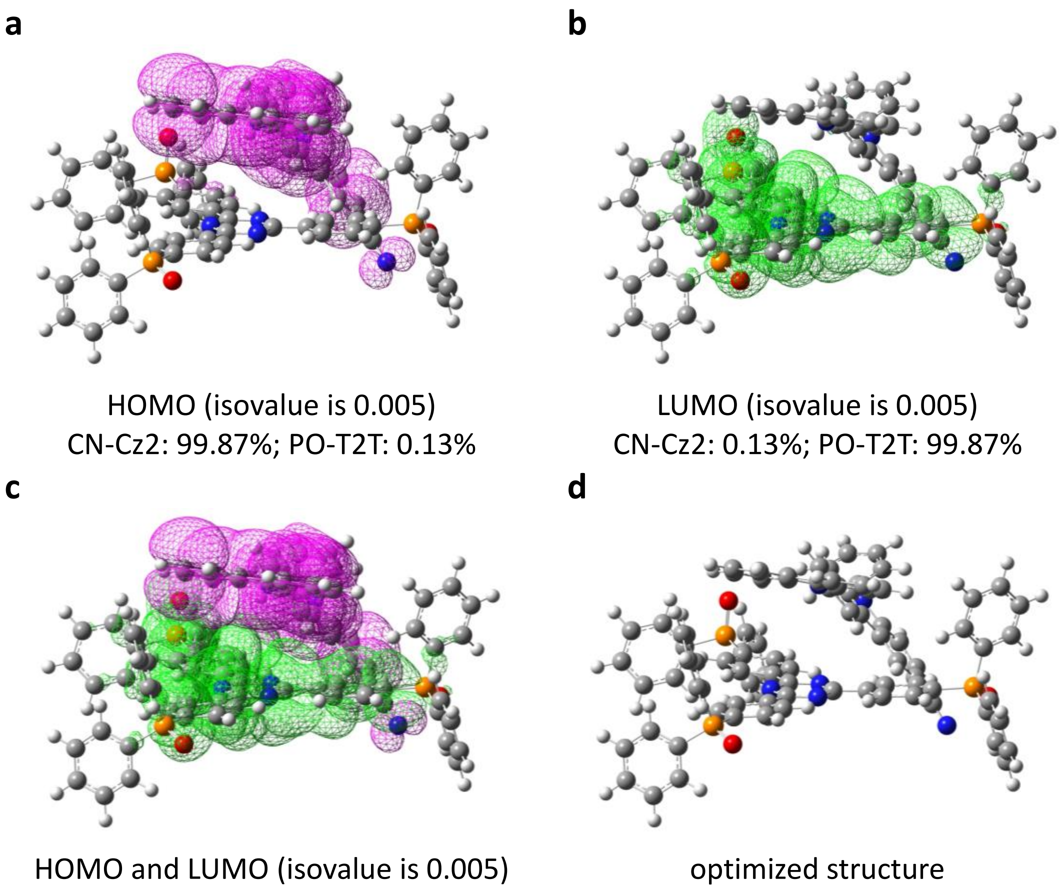

Supplementary Figure 29. Theoretical calculations based on the type I arrangement of CN-Cz2 and PO-T2T. The optimized structure of T_1 for the type I of CN-Cz2:PO-T2T exciplex in dichloromethane. The pink and green mesh are standing for (a) HOMO and (b) LUMO, respectively. The isovalue 0.02 for the coutours is defalut in GaussView. When the isolvalue is smaller (0.005), the electron density distribution “vision” for the orbital is larger. (c) The electron density distributions of HOMO and LUMO are showed. (d) The distance between the carbazole of CN-Cz2 and 1,3,5-triazine of PO-T2T is estimated around 4.38 Å**.**

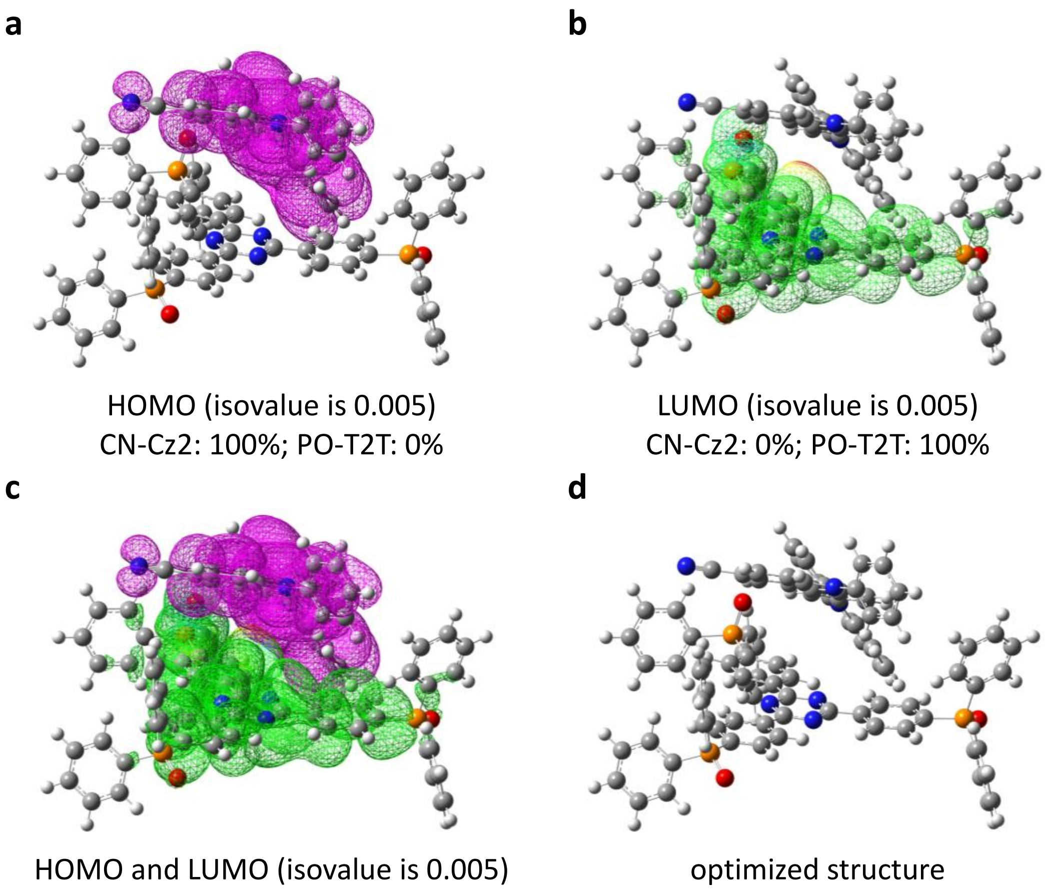

Supplementary Figure 30. Theoretical calculations based on the type II arrangement of CN-Cz2 and PO-T2T. The optimized structure of S_1 for the type II of CN-Cz2:PO-T2T exciplex in dichloromethane. The pink and green mesh are standing for (a) HOMO and (b) LUMO, respectively. The isovalue 0.02 for the coutours is defalut in GaussView. When the isolvalue is smaller (0.005), the electron density distribution "vision" for the orbital is larger. (c) The electron density distributions of HOMO and LUMO are showed. (d) The distance between the CN-carbazole of CN-Cz2 and 1,3,5-triazine of PO-T2T is estimated around 5.19 Å**.**

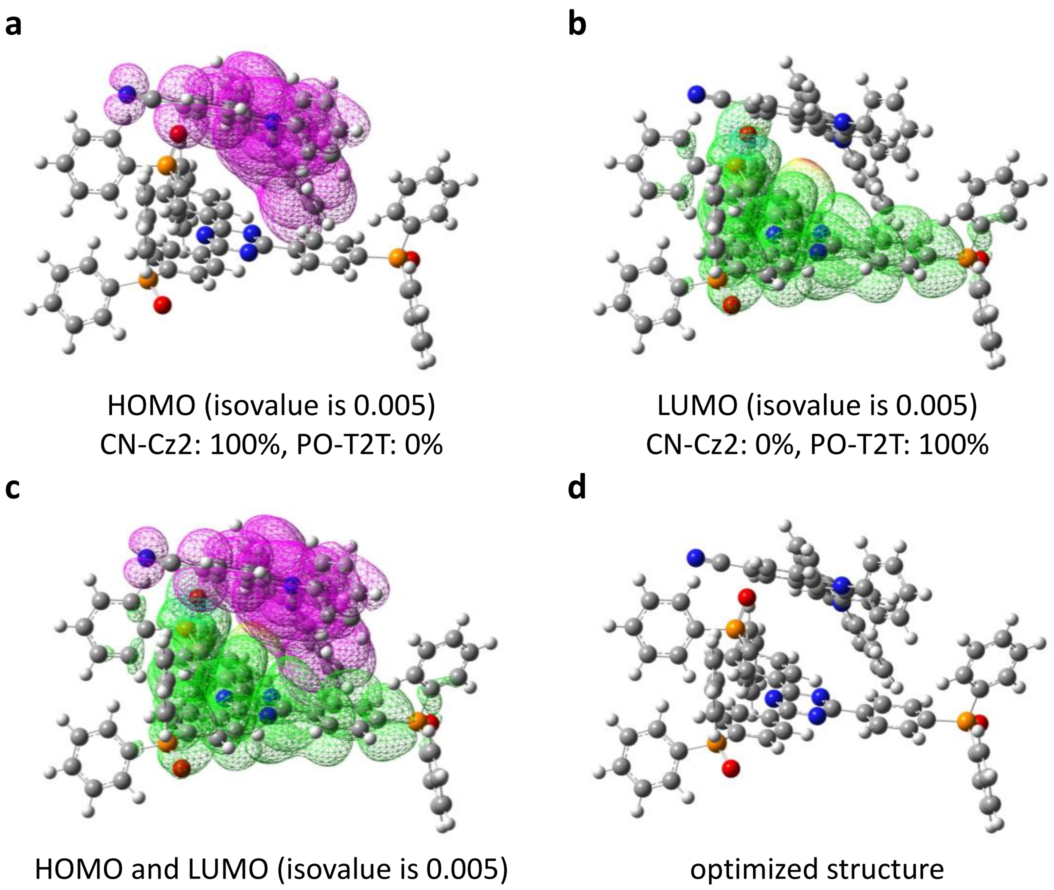

Supplementary Figure 31. Theoretical calculations based on the type II arrangement of CN-Cz2 and PO-T2T. The optimized structure of T_1 for the type II of CN-Cz2:PO-T2T exciplex in dichloromethane. The pink and green mesh are standing for (a) HOMO and (b) LUMO, respectively. The isovalue 0.02 for the coutours is defalut in GaussView. When the isolvalue is smaller (0.005), the electron density distribution "vision" for the orbital is larger. (c) The electron density distributions of HOMO and LUMO are showed. (d) The distance between the CN-carbazole of CN-Cz2 and 1,3,5-triazine of PO-T2T is estimated around 5.18 Å**.**

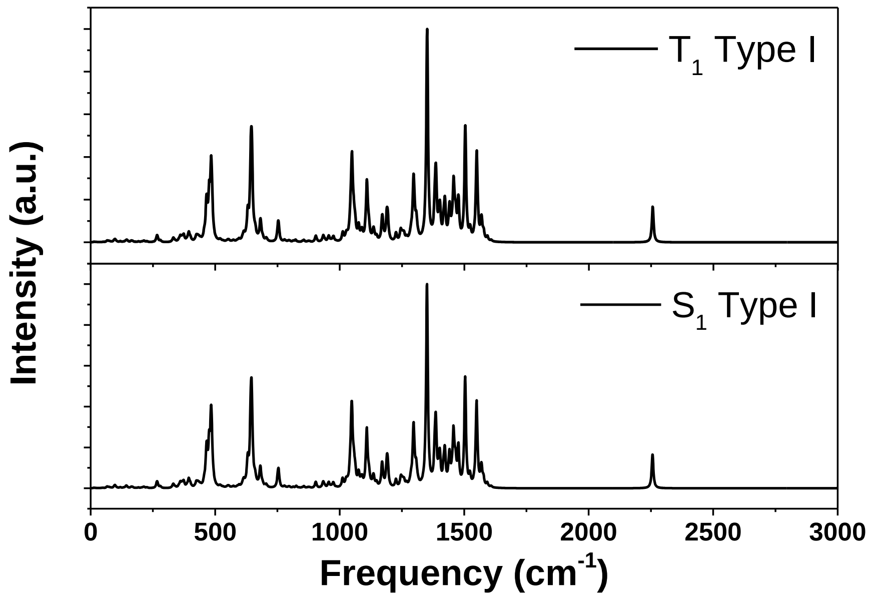

Supplementary Figure 32. The calculated IR spectra of CN-Cz2:PO-T2T exciplex with type I configurations in dichloromethane (offset by 80 cm^{-1}).

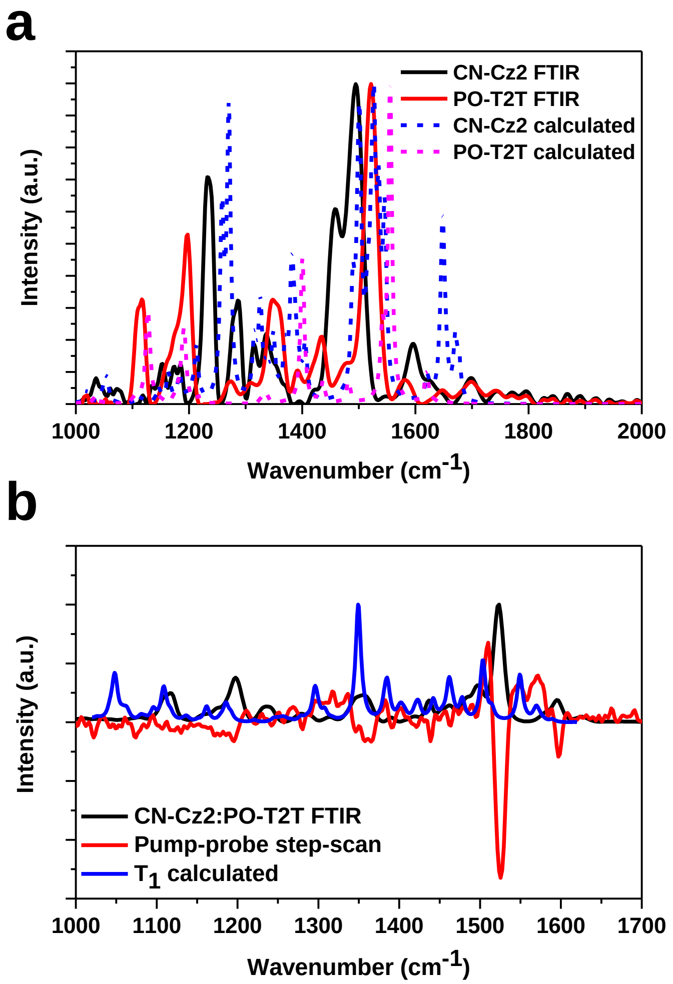

Supplementary Figure 33. Comparing the IR peaks between experiments and theoretical calculations. **a.** The experimental FTIR and calculated ground state spectra of CN-Cz2 and PO-T2T. **b.** The FTIR, pump-probe step-scan and calculated T_1 exciplex IR spectra of CN-Cz2:PO-T2T.

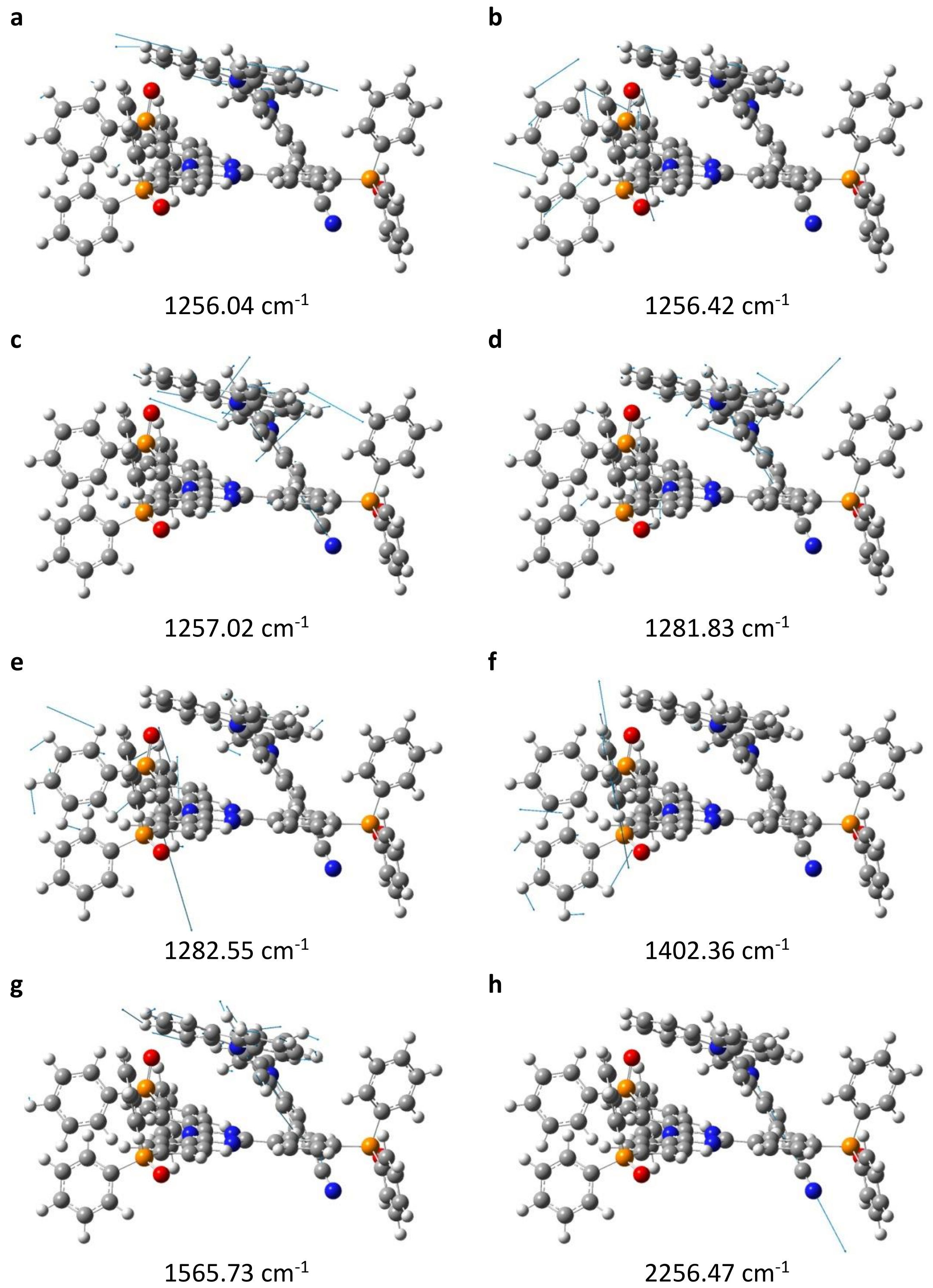

Supplementary Figure 34. Theoretical calculation of IR vibrational modes. **(a)-(g)** The calculated simultaneously IR vibrational modes for the S_1 of type I in CN-Cz2:PO-T2T exciplex within 1200 to 1600 cm^{-1} in dichloromethane. **(h)** The vibrational mode at 2256.47 cm^{-1} attributed to the –CN stretching of CN-Cz2 is also displayed.

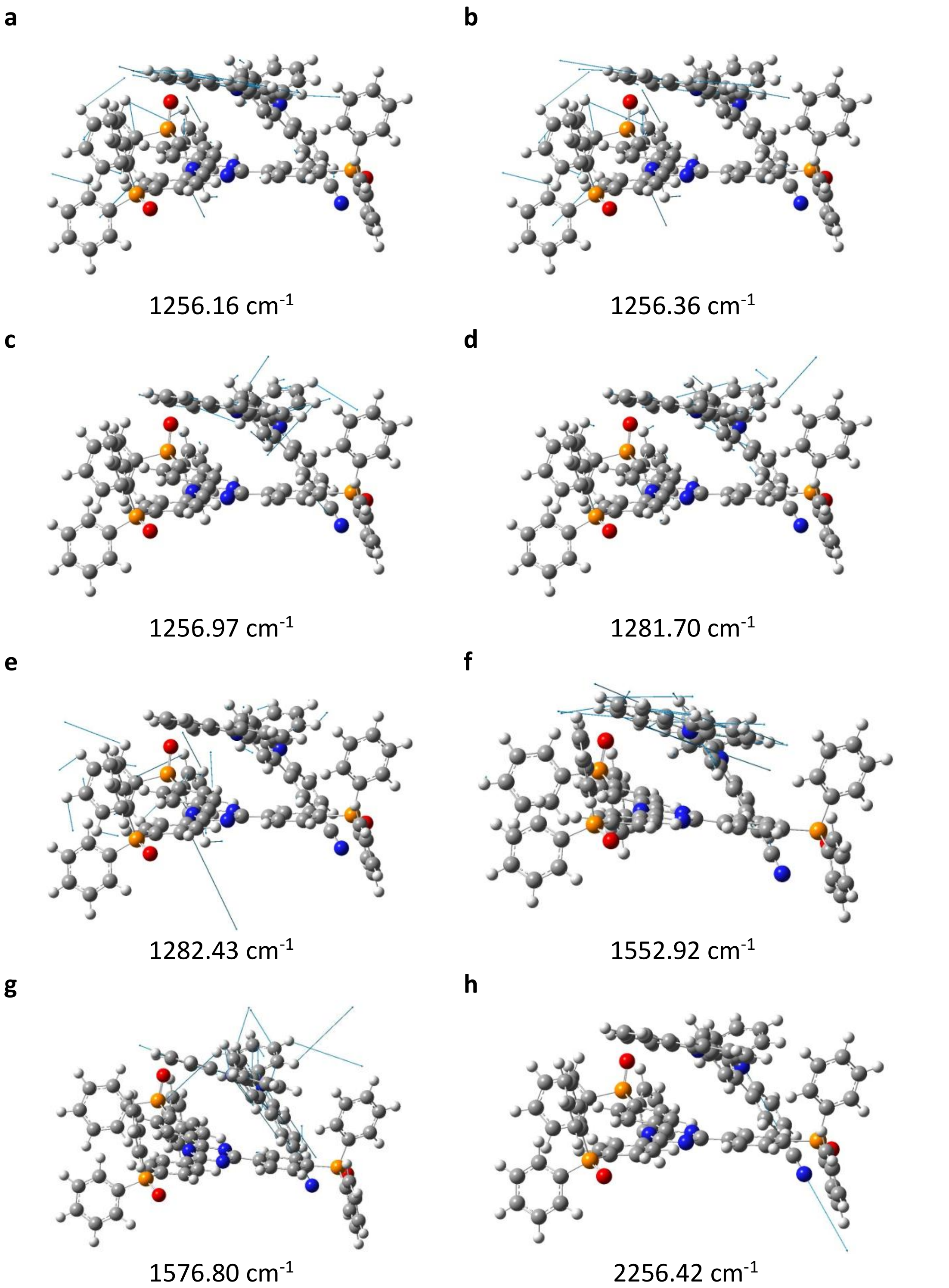

Supplementary Figure 35. Theoretical calculation of IR vibrational modes. **(a)-(g)** The calculated simultaneously IR vibrational modes for T_1 of type I in CN-Cz2:PO-T2T exciplex within 1200 to 1600 cm^{-1} in dichloromethane. **(h)** The vibrational mode at 2256.42 cm^{-1} attributed to the –CN stretching of CN-Cz2 is also displayed.

Supplementary References

1. Jiang, W.*, et al.* High-triplet-energy tri-carbazole derivatives as host materials for efficient solution-processed blue phosphorescent devices. *J. Mater. Chem.* **21,** 4918-4926 (2011).
2. Lin, M.-S.*, et al.* Incorporation of a CN group into mCP: A new bipolar host material for highly efficient blue and white electrophosphorescent devices. *J. Mater. Chem.* **22,** 16114-16120 (2012).
3. Hung, W.-Y.*, et al.* Balance the carrier mobility to achieve high performance exciplex oled using a triazine-based acceptor. *ACS Appl. Mater. Interfaces* **8,** 4811-4818 (2016).
4. Víllora, E. G., Shimamura, K., Kitamura, K. & Aoki, K. Rf-plasma-assisted molecular-beam epitaxy of β-Ga_2O_3. *Appl. Phys. Lett.* **88,** 031105 (2006).
5. Gaussian 09, Revision D.01, M. J. Frisch, G. W. Trucks, H. B. Schlegel, G. E. Scuseria, M. A. Robb, J. R. Cheeseman, G. Scalmani, V. Barone, G. A. Petersson, H. Nakatsuji, X. Li, M. Caricato, A. Marenich, J. Bloino, B. G. Janesko, R. Gomperts, B. Mennucci, H. P. Hratchian, J. V. Ortiz, A. F. Izmaylov, J. L. Sonnenberg, D. Williams-Young, F. Ding, F. Lipparini, F. Egidi, J. Goings, B. Peng, A. Petrone, T. Henderson, D. Ranasinghe, V. G. Zakrzewski, J. Gao, N. Rega, G. Zheng, W. Liang, M. Hada, M. Ehara, K. Toyota, R. Fukuda, J. Hasegawa, M. Ishida, T. Nakajima, Y. Honda, O. Kitao, H. Nakai, T. Vreven, K. Throssell, J. A. Montgomery, Jr., J. E. Peralta, F. Ogliaro, M. Bearpark, J. J. Heyd, E. Brothers, K. N. Kudin, V. N. Staroverov, T. Keith, R. Kobayashi, J. Normand, K. Raghavachari, A. Rendell, J. C. Burant, S. S. Iyengar, J. Tomasi, M. Cossi, J. M. Millam, M. Klene, C. Adamo, R. Cammi, J. W. Ochterski, R. L. Martin, K. Morokuma, O. Farkas, J. B. Foresman, and D. J. Fox, Gaussian, Inc., Wallingford CT, 2016.
6. Chen, D.-G.*, et al.* Optically triggered planarization of boryl-substituted phenoxazine: Another horizon of TADF molecules and high-performance OLEDs. *ACS Appl. Mater. Interfaces* **10,** 12886-12896 (2018).
