# Supplementary material for: Probe exciplex structure of highly efficient thermally activated delayed fluorescence organic light emitting diodes
Source: Nat Commun. 2018 Aug 6;9:3111. doi: 10.1038/s41467-018-05527-4 (PMC6079109; doi:10.1038/s41467-018-05527-4)
Supplement: Supplementary file 3 — Description of Additional Supplementary Files [file 41467_2018_5527_MOESM3_ESM.pdf]

Description of Additional Supplementary Files

File Name: Supplementary Data 1
Description: The crystallographic information file of CN-Cz2

File Name: Supplementary Data 2
Description: The checking report of the CN-Cz2 CIF file
